# Impact of Nutrition and Physical Activity Interventions Provided by Nutrition and Exercise Practitioners for the Adult General Population: A Systematic Review and Meta-Analysis

**DOI:** 10.3390/nu14091729

**Published:** 2022-04-21

**Authors:** Erin Nitschke, Kimberly Gottesman, Peggy Hamlett, Lama Mattar, Justin Robinson, Ashley Tovar, Mary Rozga

**Affiliations:** 1 Department of Exercise Science, Laramie County Community College, 1400 E College Drive, Cheyenne, WY 82007, USA; erinmd03@gmail.com; 2 Department of Kinesiology, Nutrition and Food Science, California State University Los Angeles, 5151 South University Drive, Los Angeles, CA 90032, USA; kgottes@calstatela.edu; 3 Department of Movement Sciences, University of Idaho, 875 Perimeter Drive, Moscow, ID 83844, USA; peghamlett@gmail.com; 4 Department of Natural Sciences, School of Arts and Sciences, Lebanese American University, Beirut 10150, Lebanon; lama.mattar@lau.edu.lb; 5 Kinesiology Department, Point Loma Nazarene University, 3900 Lomaland Dr, San Diego, CA 92106, USA; jrobins1@pointloma.edu; 6 Gilead Sciences, 333 Lakeside Dr, Foster City, CA 94404, USA; ashleytovar@gmail.com; 7 Evidence Analysis Center, Academy of Nutrition and Dietetics, 120 S Riverside Plaza, Suite 2190, Chicago, IL 60606, USA

**Keywords:** primary prevention, nutrition, physical activity, nutritionists, counseling, systematic review, meta-analysis, randomized controlled trial

## Abstract

Healthy dietary intake and physical activity reduce the risk of non-communicable diseases. This systematic review and meta-analysis aimed to examine the effect of interventions including both nutrition and physical activity provided by nutrition and exercise practitioners for adults in the general population (those without diagnosed disease). The MEDLINE, CINAHL, Cochrane Central, Cochrane Database of Systematic Reviews and SportDiscus databases were searched for randomized controlled trials (RCTs) published from 2010 until April 2021. Outcomes included physical activity, fruit and vegetable intake, waist circumference, percent weight loss, quality of life (QoL) and adverse events. Grading of Recommendations Assessment, Development and Evaluation (GRADE) methods were used to synthesize and grade evidence. Meta-analyses were stratified according to participant health status. The database search identified 11,205 articles, and 31 RCTs were included. Interventions increased physical activity amount [standardized mean difference (SMD) (95% CI): 0.25 (0.08, 0.43)] (low certainty evidence); increased vegetable intake [SMD (95% CI): 0.14 (0.05, 0.23)] (moderate certainty evidence); reduced waist circumference [MD (95% CI): −2.16 cm (−2.96, −1.36)] (high certainty evidence); and increased likelihood of achieving 5% weight loss for adults with overweight and obesity [relative risk (95% CI): 2.37 (1.76, 3.19)] (high certainty evidence). Very low and low certainty evidence described little-to-no effect on QoL or adverse events. Nutrition and exercise practitioners play key roles in facilitating positive lifestyle behaviors to reduce cardiometabolic disease risk in adults.

## 1. Introduction

Modifiable behaviors, such as unhealthy diet and sedentary lifestyle by physical inactivity, increase the risk of premature death from non-communicable diseases [1,2,3], which annually contribute to 71% of all deaths globally [2]. Nutrition recommendations for a healthy diet generally include individualizing intake to promote consumption of nutrient-dense foods such as vegetables and fruits, whole grains, lean proteins and healthy fats, and limit intake of added sugars, sodium, saturated fat and alcohol across the lifespan [4]. Physical activity recommendations for adults generally include performing 150 min to 300 min a week of moderate-intensity aerobic activity, or 75 min to 150 min a week of vigorous-intensity aerobic activity, or a combination of those activities. Additionally, resistance training activities focusing on all major muscle groups is recommended for adults at least two days a week [5]. Nutrition and physical activity significantly impact disease prevention; however, most adults fail to meet recommendations for the general population [1,4]. The World Health Organization (WHO) describes unhealthy diet and sedentary lifestyle by physical inactivity as leading global health risks [2].

A recent systematic review from the United States Preventive Services Task Force (USPSTF) demonstrated that behavioral interventions including both healthy diet and physical activity interventions collectively resulted in reduced risk of cardiovascular disease events and associated risk factors after 1–2 years in adults with cardiovascular disease risk [6] and can improve lifestyle behaviors and intermediate cardiometabolic outcomes in adults without cardiovascular disease risk factors [7]. Adults without diagnosed disease may have multiple risk factors such as overweight or obesity, impaired glucose tolerance, pre-hypertension, unhealthy diet, or sedentary lifestyle [8,9,10,11,12]. These adults may prefer to access allied healthcare practitioners who are available to the general population rather than seek medical care. With individualized, timely, and strategic interventions, allied healthcare practitioners can improve behaviors in adults who are healthy or have cardiometabolic risk factors to prevent disease development.

In the greater context of preventive medicine, specific allied healthcare practitioners such as registered dietitians or international equivalents (referred to as ‘dietitians’ in this manuscript), exercise practitioners, and health coaches receive unique training which positions them to enable meaningful lifestyle changes to improve health and well-being in clients. Though each of these professional groups has a distinct scope of practice [13,14,15,16], they share the common goal of facilitating lifestyle changes through nutrition and physical activity to prevent the development of cardiometabolic diseases [14,15,17]. Dietitians are credentialed nutrition practitioners who work in a variety of settings to provide quality nutrition services with an aim to improve health and well-being [13]. In contrast, exercise practitioners are certified professionals who develop safe, effective, goal-driven physical activity programs [14,15]. This two-pronged nutrition and physical activity approach to health and well-being is needed to address high population rates of unhealthy lifestyle behaviors and associated non-communicable diseases [2]. Thus, synthesized evidence is needed to determine the efficacy of nutrition and exercise practitioners in reducing cardiometabolic risk for adults prior to disease development.

The aim of this systematic review was to investigate the effects of nutrition and physical activity interventions provided by nutrition and exercise practitioners to healthy adults and those with cardiometabolic risk factors to deliver evidence-based information for practitioners and policy makers working to prevent incidence of cardiometabolic diseases. The objective of this systematic review was to examine the research question: In adults who are healthy or have cardiometabolic risk factors, what is the effect of nutrition and physical activity interventions provided by nutrition and exercise practitioners, compared to control conditions, on defined behavioral and anthropometric outcomes and quality of life?

## 2. Methods

This systematic review adhered to Grading of Recommendations, Assessment, Development and Evaluations (GRADE) methods described by the Cochrane Collaboration [18] as well as PRISMA guidelines [19] and was prospectively registered at PROSPERO (CRD42021247447) [20].

### 2.1. Eligibility Criteria

A full description of eligibility criteria can be found in Table 1. Studies were required to include adult participants (≥18 years of age) who were healthy or who had cardiometabolic risk factors, but no diagnosed disease. Cardiometabolic risk factors were overweight or obesity, and/or impaired glucose tolerance or diabetes risk or pre-hypertension, as defined by study authors. Interventions were required to include both nutrition and physical activity, last at least one month in duration, and be delivered by nutrition and/or exercise practitioners and/or health coaches. For this systematic review, nutrition practitioners were defined as registered dietitians or international equivalents [21]. Qualifying exercise practitioners were personal trainers, exercise physiologists, and those with other professional certifications recognized by the United States Registry of Exercise Professionals [22]. Health coaches were identified according to the authors’ definition. The comparison group could not receive nutrition or physical activity counseling or coaching. Outcomes of interest included: physical activity (amount and intensity), fruit and vegetable intake (measured using a validated tool), waist circumference, percent weight loss (for adults with overweight or obesity), quality of life (QoL) and adverse events. Glucose homeostasis outcomes and anxiety/depression symptoms were also examined as outcomes of interest, but results are not published in this manuscript. Randomized controlled trials (RCTs) published from January 2010 until the search date were eligible. The publication cut-off date of 2010 was selected because a recent scoping review identified several relevant articles published since this period [23] and to reflect contemporary practice. Only peer reviewed articles published in the English language were included due to resource constraints.

### 2.2. Information Sources

The full search strategy is described in Supplementary Table S1. Search strategies were written by an Information Specialist for the following databases via the Ebsco interface: Medline Complete; CINAHL Complete; Cochrane Database of Systematic Reviews; Cochrane Central Register of Controlled Trials, and SportDiscus. Searches were conducted on 2 April 2021 for articles published since 1 January 2010. Two methodological filters were used, one for systematic reviews and meta-analyses; and another for randomized controlled trials. Results were limited to the English language. Results were managed and deduplicated in Endnote Software [24]. Relevant systematic reviews were hand-searched for potentially included articles that may have been missed by the database searches.

### 2.3. Selection Process

Titles and abstracts of articles identified in the databases searched were uploaded and screened using the online Rayyan screening tool, which allows each reviewer to independently review each title and abstract and then unblind results to compare judgements with other reviewers [25]. Two reviewers independently reviewed each abstract, and discrepancies were settled using consensus or a third review. The full texts of each included title/abstract were screened by two independent reviewers to determine eligibility. Discrepancies were settled by consensus or by a third review from a content expert.

### 2.4. Data Items and Extraction

Study and intervention characteristics were extracted by trained evidence analysts and were reviewed by a lead analyst and project manager. Quantitative data were extracted by the project manager and reviewed by content experts.

Data were extracted onto a standardized template and included bibliographic information, eligibility criteria, study location and funding source, sample sizes and dropout rates, and participant characteristics (age, sex, comorbidities). Analysts also extracted information on intervention details (practitioners providing nutrition and physical activity interventions, remote vs. in-person contacts, group vs. individual contacts, number of nutrition and physical activity contacts, study duration and follow up duration, prescribed diet and physical activity) and outcomes of interest.

For outcomes measured as continuous variables, quantitative data extracted included sample size, and mean change and variance (or pre/post study mean and variance) in the intervention and control groups with an aim to calculate mean difference (MD) and 95% confidence intervals for the outcome of interest between groups. When measurement methods or units were heterogeneous, standardized mean differences (SMD) were reported. For categorical variables, the sample size and number of events were extracted for each group to calculate the relative risk (RR) of events in the intervention groups compared to the control groups. If authors reported an outcome but did not include data required for the meta-analysis, corresponding authors were contacted to request additional data. If additional data were not shared, the result was included in the narrative synthesis only.

### 2.5. Risk of Bias Assessment for Each Study

Each study was assessed for risk of bias using the updated tool for assessing RCTs from the Cochrane Collaboration, the RoB 2 tool [26]. This tool assesses risk of bias due to the randomization process, deviations from intended interventions, missing outcome data, measurement of the outcome and selection of the reported result. Each study is assigned an overall rating of “High,” “Some Concerns” or “Low” risk of bias. Risk of bias was assessed independently by two reviewers using the Cochrane Collaboration’s online algorithm tool [27]. Discrepancies in ratings for specific domains and overall ratings were settled by a third review.

### 2.6. Synthesis Methods

All studies meeting eligibility criteria and reporting at least one outcome of interest (even if full data were not available), were included in the evidence synthesis and described in the study and intervention characteristics tables. All studies reporting a particular outcome of interest were pooled using a meta-analysis when data were available. Results of studies not included in the meta-analysis were described narratively only. An overview of results for each outcome was reported on a summary of findings table, adapted from the template developed by the Cochrane Collaboration [28]. Results from risk of bias assessments were presented based on the robvis tool [29].

Meta-analyses were conducted and forest plots were created using OpenMetaAnalyst [30] and RStudio [31] software. The methodologist utilized a random-effects model to accommodate the wide range of studies included. Sensitivity analyses were conducted using leave-one-out analysis and by examining effect size according to study quality. Publication bias was described using funnel plots and Egger’s statistics. Heterogeneity was examined using the I^2^ statistic. Sub-group analyses were conducted to examine efficacy of interventions on outcomes according to whether participants were healthy or had cardiometabolic risk factors.

### 2.7. Certainty Assessment

Certainty of evidence was assessed for each outcome using the GRADE method [18,28]. Grade for certainty of evidence considered study design, number of studies and participants, risk of bias in included studies, directness of findings, precision of findings, consistency among studies, publication bias and other factors. Certainty of evidence was graded as “High,” “Moderate,” “Low,” or “Very Low” [32].

## 3. Results

### 3.1. Literature Search

**Figure 1:**
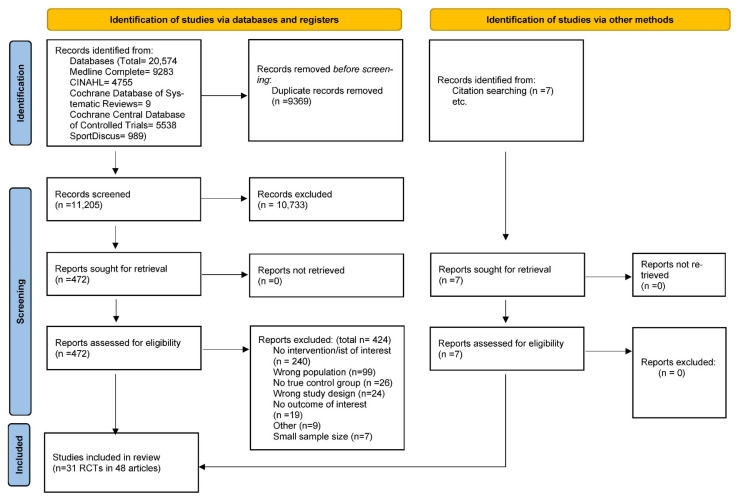
PRISMA 2020 flow diagram [19] for systematic review examining effect of nutrition and physical activity interventions provided by nutrition and exercise practitioners for the general population. *From:* Page, M.J.; McKenzie, J.E.; Bossuyt, P.M.; Boutron, I.; Hoffmann, T.C.; Mulrow, C.D.; et al. The PRISMA 2020 statement: an updated guideline for reporting systematic reviews. BMJ 2021;372:n71. doi: 10.1136/bmj.n71. For more information, visit: http://www.prisma-statement.org/ (accessed on 20 February 2022) [19].

The database search identified 11,205 unique articles; 472 full texts were reviewed, and 31 RCTs were included in this systematic review. Several studies reported results in more than one article, and, thus, forty-eight articles, describing results from the 31 RCTs, were included in this systematic review (Figure 1) [33,34,35,36,37,38,39,40,41,42,43,44,45,46,47,48,49,50,51,52,53,54,55,56,57,58,59,60,61,62,63,64,65,66,67,68,69,70,71,72,73,74,75,76,77,78,79,80].

### 3.2. Study Characteristics and Risk of Bias

Study and intervention characteristics are described in Table 2 and Table 3. Fourteen RCTs were conducted in the United States [36,38,45,49,50,51,55,59,61,65,66,69,75,78] and 17 RCTs were conducted outside of the United States [35,42,43,46,47,54,56,57,60,62,63,67,70,71,73,76,77]. Sample sizes ranged from 23 [67] to 553 [71] participants; and study durations ranged from three [43,47,50] to 48 months [54,70].

Seven RCTs targeted adults without cardiometabolic risk factors [42,43,51,57,63,66,77], while the remaining 24 RCTs targeted adults with overweight or obesity [35,36,38,45,46,47,49,50,54,55,56,59,60,62,65,67,69,73,75,76,78], diabetes risk [54,59,61,67,70,73,78], or other cardiometabolic risk factors [71]. Practitioners providing nutrition and physical activity interventions were dietitians in 12 RCTs [35,36,38,43,47,51,59,61,63,66,71,78], dietitians and exercise practitioners were combined in ten RCTs [45,46,49,50,56,57,62,67,70,75], and health coaches in six RCTs [42,55,65,69,73,77]. Three additional RCTs described dietitians that provided both nutrition and physical activity interventions and were thus included, but in these studies, their interventions included an exercise practitioner that did not meet inclusion criteria [76], an exercise practitioner was available only if requested [60], or the practitioner description was inconsistent between articles [54,72]. Exercise practitioners in included studies were primarily exercise physiologists [36,42,43,44,45,48,49,52,57,59,66,74,78,79] and trainers [55,56,61,69].

**Figure 2:**
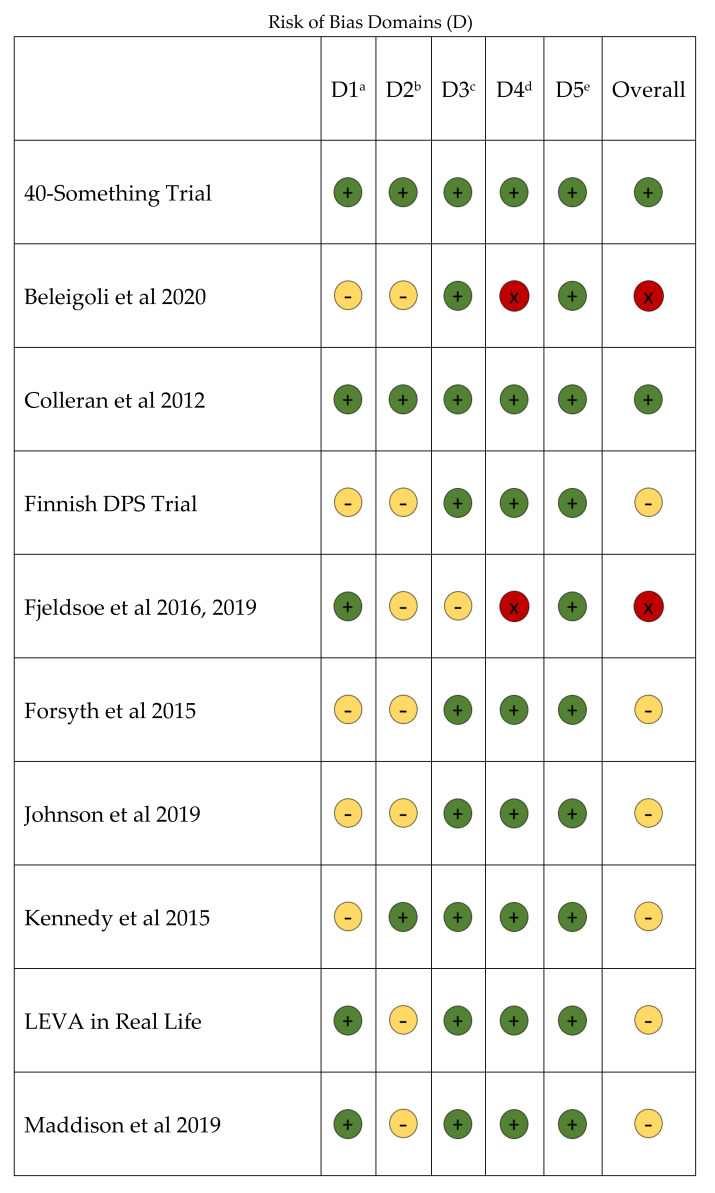

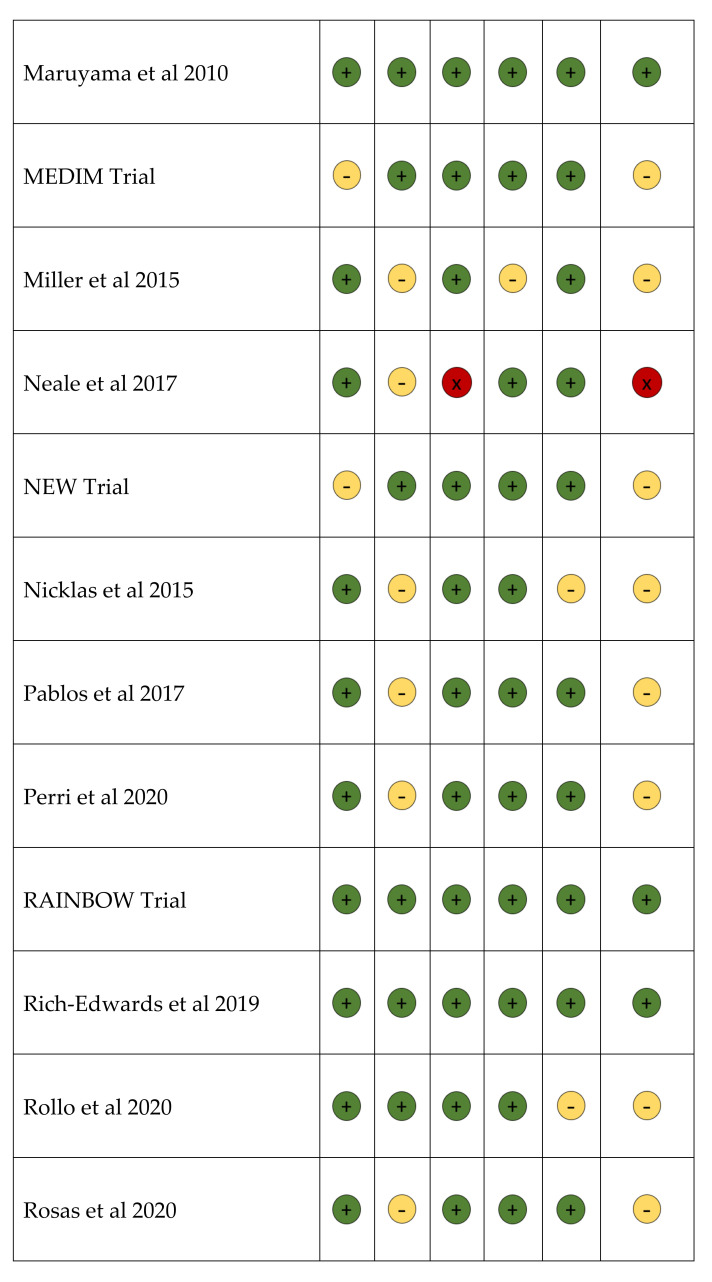

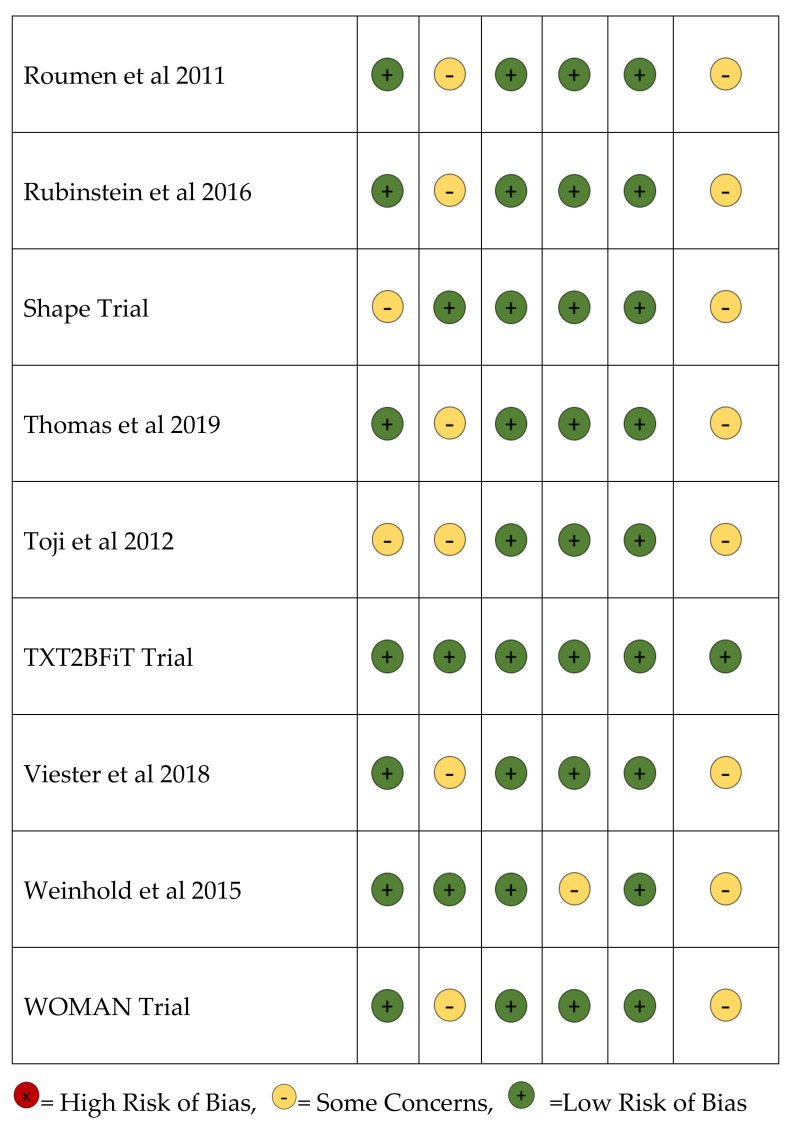
Risk of bias in the systematic review examining effect of nutrition and physical activity interventions provided by nutrition and exercise practitioners for the general population [33,34,35,36,37,38,39,40,41,42,43,44,45,46,47,48,49,50,51,52,53,54,55,56,57,58,59,60,61,62,63,64,65,66,67,68,69,70,71,72,73,74,75,76,77,78,79,80]. ^a^ D1: Bias arising from the randomization process; ^b^ D2: Bias due to deviations from intended interventions; ^c^ D3: Bias due to missing outcome data; ^d^ D4: Bias in measurement of the outcome; ^e^ D5: Bias in selection of the reported result.

The risk of bias of included RCTs is described in Figure 2. The most prevalent sources of bias were due to the randomization process, typically from lack of information regarding allocation concealment [36,43,49,50,51,54,73,76], and deviations from intended interventions and/or lack of information on intervention adherence [35,42,43,45,47,50,54,56,59,60,61,62,65,69,70,71,75,76,77]. Of the 31 included RCTs, six demonstrated Low risk of bias [38,46,55,57,63,66], 22 demonstrated Some Concerns [36,43,45,47,49,50,51,54,56,59,61,62,65,67,69,70,71,73,75,76,77,78] and three demonstrated High risk of bias [35,42,60].

A summary of findings for all included outcomes can be found in Table 4. Publication bias is described in Supplementary Figure S1.

### 3.3. Primary Outcomes

#### Physical Activity

**Figure 3:**
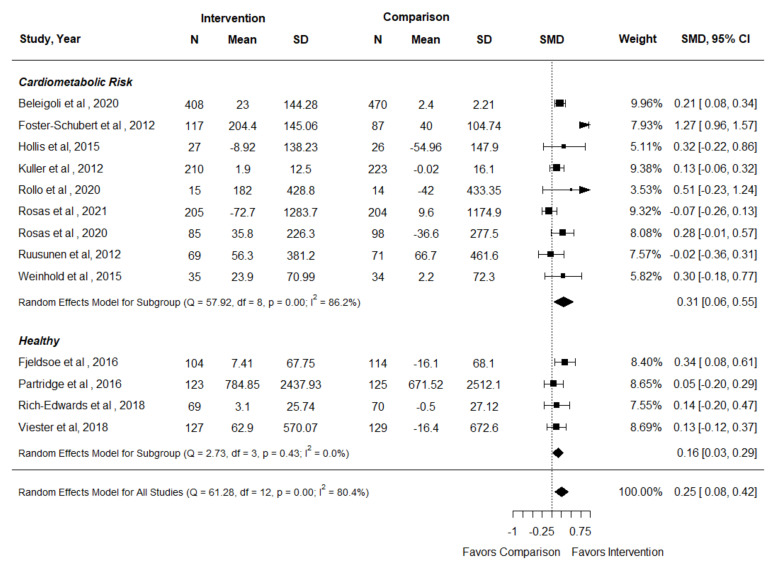
Forest plot for physical activity amount in the systematic review examining effect of nutrition and physical activity interventions provided by nutrition and exercise practitioners for the general population [35,42,44,46,53,64,66,67,68,69,72,77,78].

Seventeen RCTs represented in 22 articles examined the effect of nutrition and physical activity interventions provided by nutrition and exercise practitioners on the outcome of physical activity [34,35,41,42,44,45,46,53,56,60,61,63,64,66,67,68,69,71,72,74,77,78]. Thirteen RCTs reported quantitative data that could be pooled in a meta-analysis [35,42,44,46,53,64,66,67,68,69,72,77,78]. In a meta-analysis of 13 RCTs, the intervention resulted in a small but significant effect on physical activity amount [SMD (95% CI): 0.25 (0.08, 0.43) (I^2^ = 80.4%)] (Figure 3), and findings were significant for both participants with and without cardiometabolic risk factors. Maddison et al. reported heart rate as a measure of physical activity intensity and was not included in the meta-analysis, but the authors did report a significant reduction in resting heart rate in the intervention group compared to the control group [56]. Studies for which authors did not provide data that could be pooled in the meta-analysis reported no difference in physical activity amount between groups [60,61,71]. In adults who were healthy or had cardiometabolic risk factors, nutrition and physical activity interventions provided by nutrition and exercise practitioners may increase physical activity amount (Certainty of Evidence: Low).

### 3.4. Fruit and Vegetable Intake

Thirteen RCTs represented in 16 articles met inclusion criteria and reported the outcome of fruit and vegetable intake [34,35,39,41,42,43,46,51,59,60,63,64,68,69,71,77]. Ten RCTs reported fruit and vegetable intake separately [35,39,42,43,46,51,59,60,64,77], and three RCTs reported fruit and vegetable intake combined [68,69,71].

**Figure 4:**
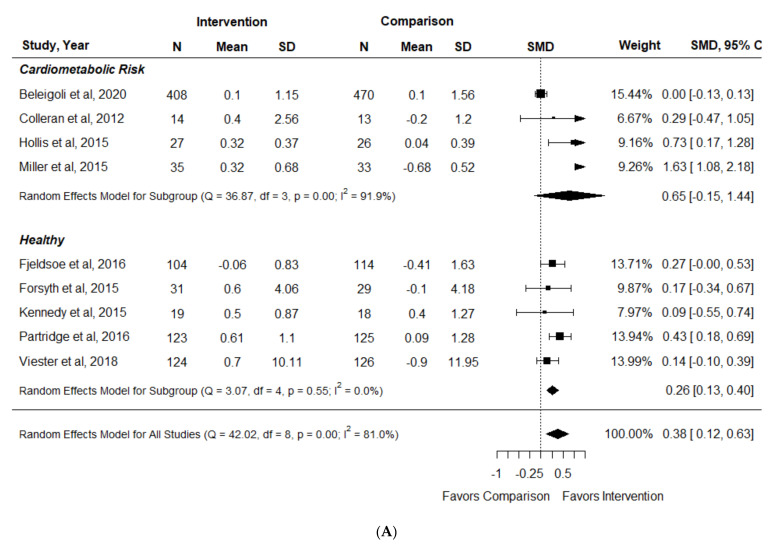

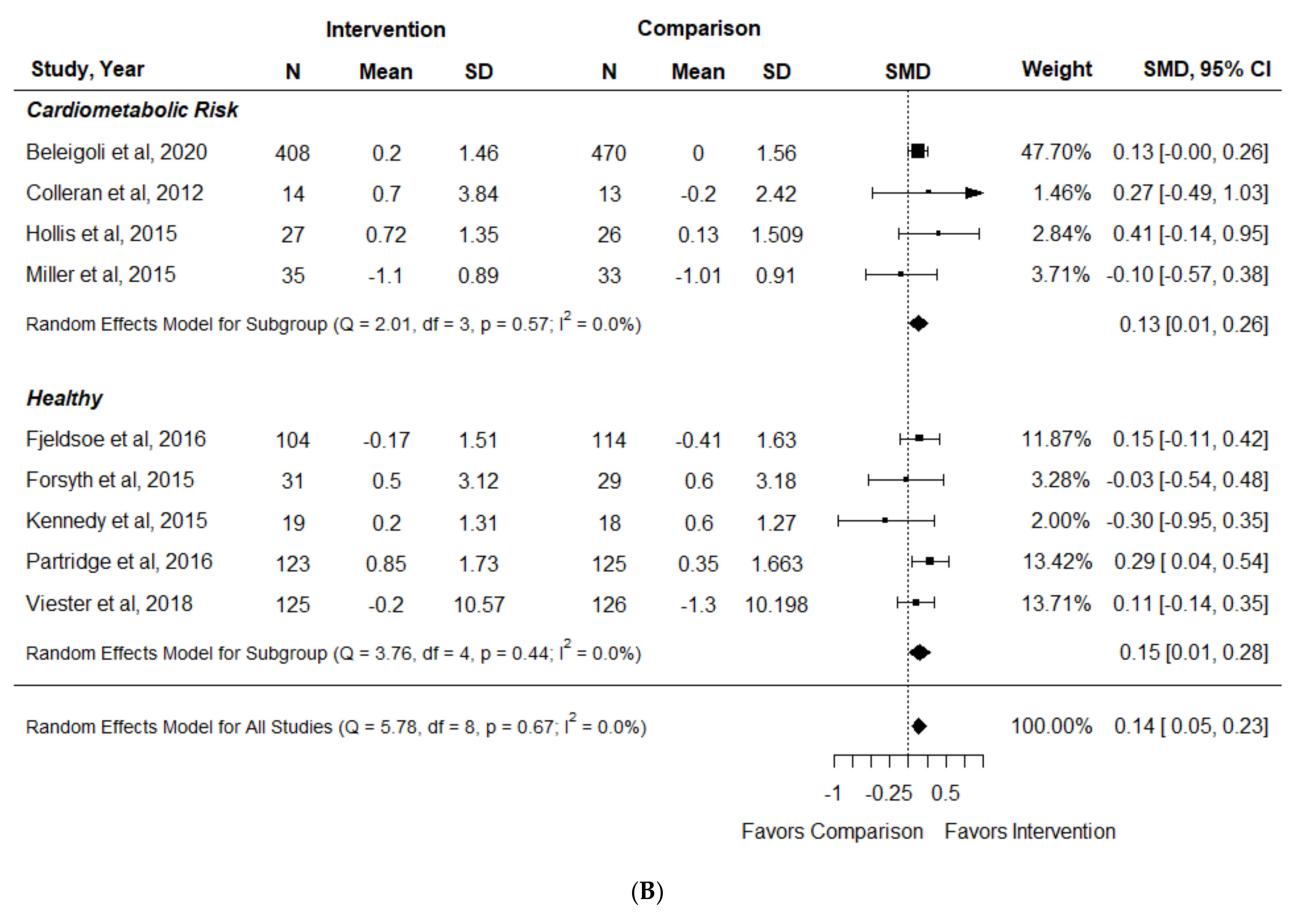
Forest plot for (**A**) fruit and (**B**) vegetable intake in the systematic review examining effect of nutrition and physical activity interventions provided by nutrition and exercise practitioners for the general population [35,39,42,43,46,51,59,64,77].

Nine of ten included RCTs reporting fruit and vegetable intake separately could be pooled in a meta-analysis [35,39,42,43,46,51,59,64,77]. In adults who were healthy, there was a small-to-moderate but significant effect of interventions on fruit intake with no heterogeneity [SMD (95% CI): 0.26 (0.13, 0.40) (I^2^ = 0%)] [42,43,51,64,77], but effect size was more heterogeneous and not significant in participants with cardiometabolic risk factors [0.65 (−0.15, 1.44) (I^2^ = 91.9%)] [35,39,46,59] (Figure 4A). Participants who were healthy experienced a small but significant increase in vegetable intake [SMD (95% CI): 0.15 (0.01, 0.28) (I^2^ = 0%)] [42,43,51,64,77], as did participants with cardiometabolic risk factors [0.13 (0.01, 0.26) (I^2^ = 0%)] [35,39,46,59] (Figure 4B). Neale et al. did not report data that could be included in a meta-analysis, but found no difference in fruit or vegetable intake between the intervention and control groups [60]. Three RCTs reported fruit and vegetable intake combined [68,69,71]. In the pooled analysis, there was no significant increase in fruit and vegetable intake in the intervention compared to control groups [SMD (95% CI): 0.10 (−0.03, 0.23) (I^2^ = 20.5%)].

In adults who are healthy, nutrition and physical activity interventions provided by nutrition and exercise practitioners increased fruit and vegetable intake, but efficacy was more heterogeneous and less certain for adults with cardiometabolic risk factors (Certainty of Evidence: Moderate).

### 3.5. Waist Circumference

**Figure 5:**
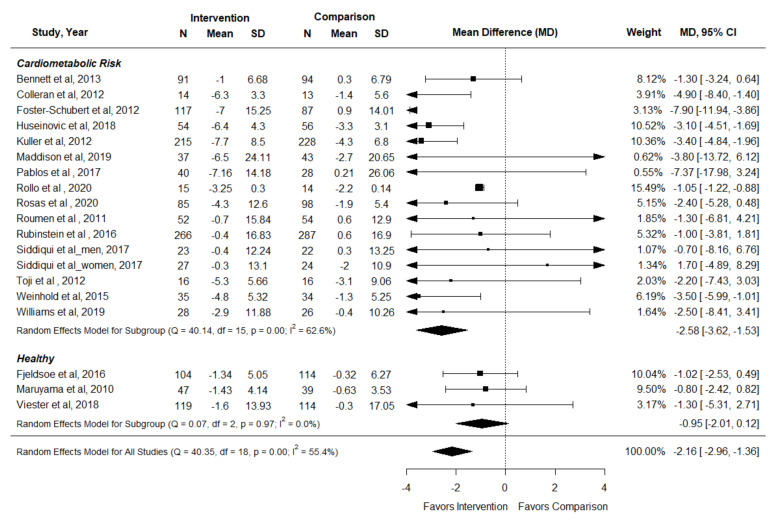
Forest plot for waist circumference in the systematic review examining effect of nutrition and physical activity interventions provided by nutrition and exercise practitioners for the general population [36,38,42,44,48,53,56,57,62,67,69,70,71,73,76,77,78,80].

Twenty-one articles representing 18 RCTs reported the effect of interventions on waist circumference [36,38,41,42,44,45,48,53,56,57,62,67,69,70,71,73,76,77,78,79,80]. All studies provided results that could be included in a meta-analysis [36,38,42,44,48,53,56,57,62,67,69,70,71,73,76,77,78,80]. In adults with cardiometabolic risk factors, nutrition and physical activity interventions from nutrition and exercise practitioners reduced waist circumference compared to control conditions across a wide range of interventions [SMD (95% CI): −2.58 cm (−3.62, −1.53) (I^2^ = 62.7%)] [36,38,44,48,53,56,62,67,69,70,71,73,76,78,80], but results were not significant in studies targeting healthy adults [−0.95 (−2.01, 0.12) (I^2^ = 0%)] [42,57,77] (Figure 5) (Certainty of Evidence: High).

### 3.6. Percent Weight Loss

*A priori*, the expert panel specified that percent weight loss would be analyzed as an outcome for participants with overweight or obesity only. Studies were required to report the number of participants achieving 5% weight loss or percent weight loss as a continuous variable.

**Figure 6:**
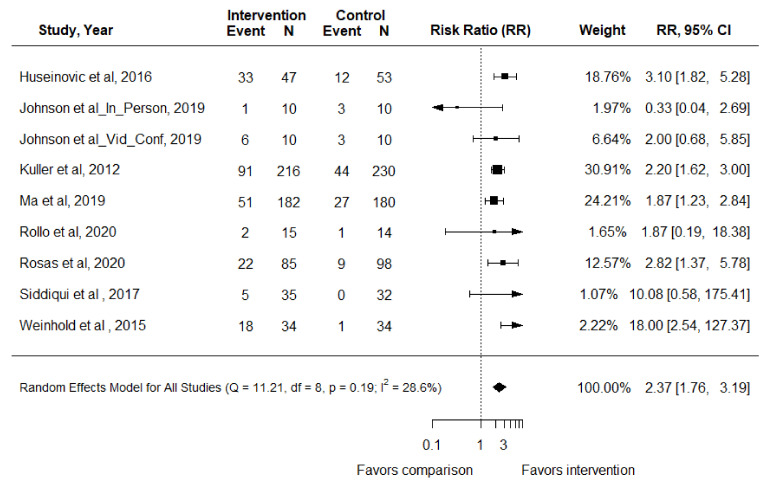
Forest plot for 5% weight loss in the systematic review examining effect of nutrition and physical activity interventions provided by nutrition and exercise practitioners for the general population [47,50,53,55,67,69,73,78].

Eight RCTs reported the outcome of achieving 5% weight loss in adults with overweight or obesity [47,50,53,55,67,69,73,78]. In the meta-analysis, adults receiving nutrition and physical activity interventions had a RR (95% CI) of 2.37 (1.76, 3.19) (*p* < 0.01) of achieving 5% weight loss compared to control groups (I^2^ = 28.6%) (Figure 6). Seven RCTs represented in nine articles reported the outcome percent weight loss as a continuous variable [33,39,40,50,54,58,59,75,78]. In a pooled analysis of three RCTs, there was no significant effect of interventions on percent weight loss [MD (95% CI): −2.37% (−5.51, 0.77) (I^2^ = 79.9%)] [50,75,78]. In the remaining four studies, authors did not provide variance to the mean weight loss percentages reported, but all reported increased percent weight loss in participants who received the interventions compared to the controls [39,54,58,59].

*Post-hoc*, authors investigated if percent weight loss results varied according to if authors described caloric reduction as part of the intervention. Of the 13 RCTs that reported percent weight loss as an outcome for adults with overweight or obesity [39,47,50,53,54,55,58,59,67,69,73,75,78], authors of ten studies described that caloric reduction was advised and participants experienced significant percent weight loss or increased likelihood of reaching 5% weight loss in nine of these ten RCTs [39,47,53,54,55,58,59,69,78], but no effect in one RCT [75]. Three RCTs that did not describe prescribed caloric reduction did not result in significant percent weight loss [50,67,73].

In adults with overweight or obesity but no diagnosed disease, nutrition and physical activity interventions from nutrition and exercise practitioners improved likelihood of achieving 5% weight loss compared to controls, but there was no effect on percent weight loss as a continuous variable. Percent weight loss was generally only significant compared to controls when caloric reduction was prescribed (Certainty of Evidence: Moderate).

### 3.7. Quality of Life

Seven included RCTs reported the outcome of QoL [49,51,52,60,67,69,80]. Study authors used a variety of tools to measure QoL. Because three RCTs utilized the Short Form-36 (SF-36), a common tool for determining QoL, these studies were included in the meta-analysis [49,51,80] and demonstrated no significant effect in the intervention groups compared to control groups on SF-36 Physical QoL [MD (95% CI): 3.91 (−0.21, 8.03) (I^2^ = 57.3%)] or Mental QoL [0.19 (−4.04, 4.42) (I^2^ = 33.1%)]. In Rosas et al., an Obesity-related Problems Scale was used to measure QoL, in which a lower number is a better outcome. There was no difference in outcomes between groups [69]. In a study by Krishnan et al., transformed weight was used as a proxy for QoL, and there was a greater increase in QoL in the intervention group, but there was no statistical comparison between groups [52]. When the Assessment of QoL 6-dimension tool was used in a small sample, QoL was improved in the intervention group compared to the control group [67]. In a study by Neale et al., the authors used the Short Form-12 to measure QoL and results were reported as medians (interquartile range). The authors reported no difference in QoL between groups [60].

In adults who are healthy or have cardiometabolic risk factors, the evidence is very uncertain about the effect of nutrition and physical activity interventions provided by nutrition and exercise practitioners on physical and mental quality of life but suggests little-to-no effect (Certainty of Evidence: Very Low).

### 3.8. Adverse Events

Three included RCTs reported adverse events [37,55,69]. In Ma et al. [55] and Rosas et al. [69], serious and nonserious adverse events were comparable between intervention and control groups. However, in a study targeting postmenopausal women, musculoskeletal injuries and hot flash number as well as severity were not significantly different between groups, but bone mineral density was decreased in the diet and exercise group compared to the control group (−1.7% compared with 0% change in the control group) [37]. In adults who were healthy or had cardiometabolic risk factors, nutrition and physical interventions provided by nutrition and exercise practitioners may result in little to no difference in adverse events, though postmenopausal women in an intervention group had reduced bone mineral density compared to the control group in one study (Certainty of Evidence: Low).

## 4. Discussion

The results of this systematic review demonstrate that combined nutrition and physical activity interventions provided by nutrition and exercise practitioners may increase physical activity amount (low certainty of evidence) and fruit and vegetable intake (low-to-moderate certainty of evidence), decrease waist circumference (high certainty of evidence), and improve the likelihood of achieving a 5% weight loss for adults with overweight or obesity (high certainty of evidence). Interventions may result in little to no difference in QoL (very low certainty of evidence), and adverse events (low certainty of evidence). The results demonstrated that interventions were more effective for fruit intake among healthy adults and were more effective for anthropometric outcomes among adults with cardiometabolic risk factors.

The evidence from this systematic review is consistent with findings from similar reviews. A 2020 systematic review conducted by the USPSTF demonstrated that medium- and high-contact multisession behavioral coaching nutrition and physical activity interventions were effective in reducing cardiovascular events, lowering blood pressure, and improving blood lipid levels in adults with cardiovascular risk factors [6]. A systematic review by Abbate et al. similarly demonstrated beneficial effects of diet and physical activity training in adults with cardiometabolic risk factors [81]. For adults without cardiovascular risk factors, findings from the current systematic review aligned with those from a 2022 systematic review by the USPSTF that nutrition and physical activity interventions improved dietary intake and physical activity amount [7]. Other systematic reviews have focused on the effectiveness of nutrition or physical activity interventions alone. For example, a 2021 systematic review by Jinnette et al. found that personalizing nutrition advice improved dietary intake compared to generalized nutrition advice [82], which supports the need for individualized client counseling. The current systematic review is unique because it specifically considers the effect of interventions including both nutrition and physical activity provided by nutrition and exercise health practitioners and targets participants who may be at risk for cardiometabolic disease due to poor lifestyle behaviors or cardiometabolic risk factors. This focus is important because clients can access such allied healthcare practitioners outside of traditional clinical and medical organizations. In addition, it provides policy makers with information on specific means (nutrition and exercise practitioners) to deliver effective interventions for disease prevention. The evidence from this and other current systematic reviews supports the importance and efficacy of early interventions to reduce cardiometabolic disease risk.

Interestingly, the results of this review revealed little to no effect on QoL. A 2021 systematic review by Jones et al. demonstrated that behavioral weight management interventions improved mental QoL and reduced depression [83], and a 2021 systematic review by the Academy of Nutrition and Dietetics found that overweight and obesity treatment interventions provided by a dietitian improved physical and mental QoL [84]. Conclusions related to the impact that lifestyle interventions have on psychosocial outcomes are, at this point, uncertain, and specific lifestyle interventions that improve QoL, particularly mental QoL, are unknown.

The efficacy of nutrition and physical activity interventions demonstrated in this review is encouraging. However, it is important to note and recognize the varying scopes of practice for each nutrition and exercise allied health practitioner, including when it may be appropriate for a practitioner to give general health recommendations outside of their area of expertise and when it is appropriate to refer to another allied health practitioner.

An allied health practitioner’s nutritional scope of practice is determined by a combination of national certification and credentialing [85], state laws and regulations, and the professional’s education, experience, and skillset. Thus, a high degree of variability among different practitioners exists regarding what advice and interventions they can ethically provide when dispensing nutrition and physical activity guidance and designing interventions. For example, dietitians have a wider and more sophisticated scope of practice as it relates to nutrition, medical nutrition therapy, nutrient analysis, and individualized meal planning compared to an exercise practitioner or health coach [13,16]. An exercise practitioner, such as a certified personal trainer and/or health coach, has a more limited scope with respect to nutrition, and would likely benefit from referring clients with metabolic risk factors, such as obesity, to dietitians. However, exercise professionals may discuss certain aspects of nutrition with clients. Exercise practitioners, including health coaches, who have earned an accredited certification [85] can and should educate clients and discuss the following: principles of healthy nutrition and food preparation, characteristics of a balanced diet, essential nutrients, actions of nutrients, effects of deficiencies and excess of nutrients, nutrient requirements throughout the lifespan, principles of pre and post-workout fueling, and information about nutrients in foods or supplements [15]. Certified health coaches, more specifically, can apply effective communication skills to assist clients in taking ownership of their behavior changes. Additionally, health coaches support and empower clients to develop measurable goals and the internal strength to achieve those goals [14].

Alternatively, it may be appropriate for dietitians or health coaches to provide generalized physical activity guidance to adults who are apparently healthy or who do not have physical activity limitations [13,16]. However, in more complex cases such as when clients have limited mobility due to obesity, limited experience with physical activity, or highly specific physical activity goals such as building muscle mass, referral to exercise practitioners may be warranted. Multidisciplinary collaboration allows allied health practitioners to share expertise, provides a trustworthy system for client referrals and increases access to interventions to empower adults to prevent disease.

## 5. Strengths and Limitations

Strengths of this systematic review and meta-analysis included rigorous methods that adhered to GRADE and PRISMA standards. In addition, included studies examined a wide range of nutrition and physical activity interventions provided by a variety of nutrition and exercise practitioners. This systematic review was conducted using a multidisciplinary team of researchers and practitioners in the fields of nutrition, physical activity, and behavior change. Finally, this meta-analysis examined multiple outcomes of interest that are commonly collected in practice and important to population health.

A limitation of this systematic review was that the limited number of studies for each outcome and the heterogeneous interventions and results prevented the team from drawing generalizable conclusions regarding the efficacy of specific types of interventions that include nutrition and physical activity, such as delivering intervention using telehealth or in a group setting. The GRADE method specifies that the number of outcomes selected for analysis are limited to seven outcomes, thus limiting examination of other important healthy dietary components such as intake of whole grains and added sugars. Further, this review relied on exclusively peer-reviewed literature, and there is the potential that unpublished, but applicable, literature relevant to the research question was not included. Finally, this review does not include an intentional analysis of specific sub-populations who are at higher risk for cardiometabolic disease, such as those with low socioeconomic status or who identify as members of racial or ethnic minority groups. Evidence for some outcomes was limited by the risk of bias of included studies or by the lack of studies reporting the outcome of interest, such as QoL.

## 6. Future Research

Future research should aim to investigate the effects of nutrition and physical activity interventions in underserved populations, such as those with low socioeconomic status or who identify as members of racial or ethnic minority groups, and others who are at higher risk for developing non-communicable diseases. Another goal of future research is to examine nutrition and physical activity interventions that specifically investigate a behavior change-based approach compared to education/information only based interventions. Investigations comparing process (behavior) goals versus product (outcome) goals would provide significant value. A third goal of future research is to examine the optimal number of sessions, types of interactions (e.g., one-on-one vs. group or in-person vs. remote), and/or number of contacts between a client and nutrition and/or exercise practitioners for effectively eliciting behavior change and positive habit development that promote sustainable and meaningful lifestyle changes.

## 7. Conclusions

Recent research demonstrates that allied health practitioners including dietitians, exercise practitioners and health coaches may facilitate improvement of lifestyle behaviors and anthropometric outcomes, and thus play a key role in improving population health by collaborating with clients who are healthy or who have cardiometabolic risk factors to reduce disease risk. However, more research is needed to determine consistent and effective delivery of interventions to a diversity of clients. Adults would benefit from improved access to allied health practitioners prior to disease development to establish healthy lifestyle behaviors through encouragement, education and skill development. Complementary practitioners can team up to provide multidisciplinary, comprehensive services to their clients while staying within their scopes of practice.

## Figures and Tables

**Table 1:** Eligibility criteria for systematic review examining the effect of nutrition and physical activity interventions provided by nutrition and exercise practitioners.

Category	Inclusion	Exclusion
Setting	Community, work, university, research and other “public” settings, primary care settings	In-patient
Population	Humans Adults ≥ 18 years of age Health Status: Healthy or with cardiometabolic risk factors (including overweight or obesity, pre-diabetes and pre-hypertension) but no diagnosed disease. Studies targeting women who are postpartum/lactating are included	Animal studies <18 years of age Professional or elite athletes Family is the target population Health Status: Any diagnosed disease or conditions limiting generalizability to individuals in the general population including but not limited to: Type 2 diabetes mellitus Cardiovascular disease Non-alcoholic fatty liver disease Chronic kidney disease Cancer Eating disorders Chronic obstructive pulmonary disorder Human immunodeficiency virus infection and acquired immune deficiency syndrome Heart failure, stroke Post-bariatric surgery Severe or persistent mental illness Hypertension Dyslipidemia Metabolic syndrome Frail elderly Osteoarthritis Pregnancy Diagnosed sleep apnea Cognitive impairment
Intervention	Must include nutrition AND physical activity Multi-disciplinary beyond nutrition and physical activity are included (e.g., includes intervention from behavioral therapist, nurse, etc.)	Only includes nutrition OR physical activity
Intervention Provider	Interventions delivered by a dietitian or international equivalent, exercise practitioner (see below), or health coach Exercise practitioners as defined by United States Registry of Exercise Professionals http://usreps.org/Pages/credentials.aspx (accessed on 20 February 2022) [22] If the interventionist was defined as a “nutritionist”, the authors checked the following website to determine if this was a dietitian equivalent in the country of interest or emailed the corresponding author: https://www.internationaldietetics.org/NDAs.aspx (accessed on 20 February 2022) [21] “Health Coaches” were identified according to the author’s definition.	Interventions provided by professionals not specified in inclusion. Practitioner delivering the intervention is not specified. Interventions provided by lifestyle coaches Health coaches
Intervention Duration	≥1 month	<1 month
Control and Comparison Groups	Control group for the overarching question is no intervention, wait list, or other control that is not a nutrition or exercise intervention. Comparisons defined in sub-questions are investigated with sub-analyses (ex: efficacy of interventions delivered by telehealth (vs control) compared to efficacy of interventions delivered in-person (vs control)).	Comparison group receives the same level of nutrition and/or physical activity intervention compared to the intervention group.
Outcomes	Quality of life, anxiety/depression, physical activity (exercise duration (ex: min/week) or intensity measured as heart rate, rated perceived exertion or metabolic equivalents, fruit and vegetable intake (measured using a validated tool), waist circumference, percent weight loss (measured as a continuous variable for those with overweight/obesity or as proportion of participants achieving 5 percent weight loss)	Outcomes not defined in inclusion criteria.
Study Design	Randomized controlled trials Relevant systematic reviews and meta-analyses are searched for potentially included articles missed by the database search.	Non-randomized trials, non-controlled trials, observational studies, commentaries, narrative reviews.
Sample size	≥10 in each group	<10 in each group
Year	January 2010–2 April 2021	Prior to January 2010 or after the search date of 2 April 2021
Publication	Peer-reviewed publications.	Grey literature, conference abstracts
Language	Articles published in the English language.	Articles published in languages other than English.
Databases Searched	MEDLINE, CINAHL, SportsDiscus, Cochrane Database of Systematic Reviews, Cochrane Database of Controlled Trials	-

**Table 2:** Study characteristics of randomized controlled trials included in the systematic review examining effect of nutrition and physical activity interventions provided by nutrition and exercise practitioners for the general population.

Trial Name (If Applicable), Author, Year	Country	Setting	Target Population	Mean Age (Years)	Sample Size (Final N)	Duration (Months)	Funding Source	Risk of Bias
**40-Something Trial** Hollis et al. 2015 [46] Williams et al. 2014 [79] Williams et al. 2019 [80]	Australia	Research/University	Female adults (44–50 years) with healthy weight or overweight (BMI = 18.5–29.9 kg/m^2^)	Intervention: 47.6 Control: 46.9	40	12	University/Hospital	Low Risk
Beleigoli et al. 2020 [35]	Brazil	Research/University	Adults with overweight or obesity (BMI ≥ 25 kg/m^2^)	Intervention (mean): 33.0 Control: 33.4	473	6	Government	High Risk
Colleran et al. 2012, [38,39]	United States	Community	Female adults with overweight or obesity (BMI 25–30 kg/m^2^), postpartum	Intervention: 30.3 Control: 31.9	27	4	Government, University/ Hospital	Low Risk
**Finnish DPS Trial** Lindstrom et al. 2013 [54] Ruusunen et al. 2012 [72]	Finland	Outpatient/Primary Care	Adults with type 2 diabetes risk, overweight or obesity (BMI ≥ 25 kg/m^2^)	Intervention: 55 Control: 55	480	48	Government, University/Hospital, Not-for-profit	Some Concerns
Forsyth et al. 2015 [43]	Australia	Outpatient/Primary Care	Adults with anxiety/depression (Mean BMI 31.6 and 31.8 kg/m^2^ for Intervention and Control)	NR	94	3	Government	Some Concerns
**GHSH Trial** Fjeldsoe et al. 2016 [42] Fjeldsoe et al. 2019 [41]	Australia	Community	Adults (Mean BMI 29.5 kg/m^2^)	Intervention: 55.5 Control: 51.2	211	6	Government, University/Hospital	High Risk
Johnson et al. 2019 [50]	United States	Research/University	Adults with obesity (BMI ≥ 30 kg/m^2^)	Intervention: 42.2 Control: 44.5	20	3	Government, Industry	Some Concerns
Kennedy et al. 2015 [51]	United States	Community	Adults identified as African American (BMI ≥ 23 kg/m^2^)	Intervention: 54 Control: 54	37	12	Not-for-profit	Some Concerns
**LEVA in Real Life Trial** Huseinovic et al. 2016 [47] Huseinovic et al. 2018 [48]	Sweden	Outpatient/Primary Care	Female adults with overweight or obesity (BMI ≥ 27 kg/m^2^), postpartum	Intervention: 31.8 Control: 32.6	89	3	Government, Not-for-profit	Some Concerns
Maddison et al. 2019 [56]	New Zealand	Community	Male adults with overweight or obesity (BMI ≥ 25 kg/m^2^)	Intervention: 40.6 Control: 44.7	80	4	NR	Some Concerns
Maruyama et al. 2010 [57]	Japan	Community	Adults (Mean BMI 25.7 and 25.8 for Intervention and Control)	Intervention: 43.1, 7.7 Control: 35.5, 8.1	87	4	Not-for-profit	Low Risk
**MEDIM Trial** Siddiqui et al. 2017 [73] Siddiqui et al. 2018 [74]	Sweden	Research/University	Adults with type 2 diabetes risk, overweight or obesity (BMI ≥ 28 kg/m^2^)	Intervention: 47.9 Control: 48.9	67	4	Industry, University/Hospital	Some Concerns
Miller et al. 2015 [59]	United States	Research/University	Adults with type 2 diabetes risk (no information on BMI)	Intervention: 51.6 Control: 50.8	68	4	Government	Some Concerns
Neale et al. 2017 [60]	Australia	Community	Adults with overweight or obesity (BMI ≥ 25–40 kg/m^2^)	Intervention: 43.79 Control: 42.10	189	12	Industry, Government, Not-for-profit	High Risk
**NEW Trial** Abbenhardt et al. 2013 [33] Campbell et al. 2012 [37] Duggan et al. 2016 [40] Foster-Schubert et al. 2012 [44] Imayama et al. 2011 [49] Mason et al. 2011 [58]	United States	Research/University	Female adults with overweight or obesity (BMI ≥ 25 kg/m^2^)	Intervention: 58.0 Control: 57.4	188	12	Government	Some Concerns
Nicklas et al. 2014 [61]	United States	Community	Female adults with type 2 diabetes risk, postpartum (Mean BMI 31.2 and 31.6 kg/m^2^ in the Intervention and Control groups)	Intervention: 33.6 Control: 33.3	71	12	Government	Some Concerns
Pablos et al. 2017 [62]	Italy	Research/University	Adults with overweight or obesity (BMI ≥ 25 kg/m^2^)	Intervention: 49.80 Control: 51.25	68	8	University/Hospital	Some Concerns
Perri et al. 2020 [65]	United States	Community	Adults with obesity (BMI 35–40 kg/m^2^)	Intervention: 55.9 (individual counseling) and 55.4 (group counseling) Control: 54.8	260	6	Government	Some Concerns
**RAINBOW Trial** Ma et al. 2019 [55] Rosas et al. 2021 [68]	United States	Outpatient/Primary Care	Adults with overweight or obesity (BMI ≥ 30 kg/m^2^ or ≥27 kg/m^2^ if Asian)	Intervention: 50.9 Control: 51.0	371	12	Government	Low Risk
Rich-Edwards et al. 2019 [66]	United States	Community	Adults, postpartum (BMI ≥ 18.5–40 kg/m^2^)	Intervention: 30.5 Control: 31.7	139	9	Government	Low Risk
Rollo et al. 2020 [67]	Australia	Community	Female adults with risk of type 2 diabetes, overweight or obesity (BMI ≥ 18.5–50 kg/m^2^), postpartum	Intervention: 34.0 Control: 33.6	23	6	Not-for-profit	Some Concerns
Rosas et al. 2020 [69]	United States	Outpatient/Primary Care	Adults with overweight or obesity (BMI ≥ 24 kg/m^2^)	Intervention: 50.3 Control: 50.1	183	12	Government	Some Concerns
Roumen et al. 2011 [70]	Netherlands	Research/University	Adults with type 2 diabetes risk (Mean BMI 29.9 kg/m^2^ and 29.7 kg/m^2^ for Intervention and Control groups)	Intervention: 55.0 Control: 58.8	109	48	Government, Not-for-profit	Some Concerns
Rubinstein et al. 2016 [71]	Argentina, Guatemala, Peru	Community	Adults with pre-hypertension (Mean BMI 30.2 kg/m^2^ and 30.8 kg/m^2^ for Intervention and Control groups)	Intervention: 48.6 Control: 43.2	553	12	Government, Industry	Some Concerns
**Shape Trial** Bennett et al. 2013 [36] Krishnan et al. 2019 [52]	United States	Outpatient/Primary Care	Female adults with overweight or obesity (BMI 25–34.9 kg/m^2^)	Intervention: 35.6 Control: 35.2	177	12	Government	Some Concerns
Thomas et al. 2019 [75]	United States	Research/University	Adults with overweight or obesity (BMI 25–45 kg/m^2^)	NR	125	18	Government	Some Concerns
Toji et al. 2012 [76]	Japan	Community	Adults with overweight or obesity (BMI 24–28 kg/m^2^)	Intervention: 61 Control: 62	32	6	Government	Some Concerns
**TXT2BFiT Trial** Allman-Farinelli et al. 2016 [34] Partridge et al. 2015 [63] Partridge et al. 2016 [64]	Australia	Telehealth	Adults at risk of weight gain (BMI 23–32 kg/m^2^)	18–35	248	9	Government, Not-for-profit	Low Risk
Viester et al. 2018 [77]	Netherlands	Workplace	Male adults (Mean BMI 27.4 kg/m^2^)	Intervention: 46.3 Control: 47.0	277	6	Foundation	Some Concerns
Weinhold et al. 2015 [78]	United States	Workplace, Research/University	Adults with type 2 diabetes risk, overweight or obesity (BMI 25–50 kg/m^2^)	Intervention: 51.6 Control: 51.0	67	4	Government	Some Concerns
**WOMAN Trial** Gabriel et al. 2011 [45] Kuller et al. 2012 [53]	United States	Research/University	Female adults with overweight or obesity (BMI 25–39.9 kg/m^2^)	Intervention: 56.9 Control: 57.1	400	36	Government	Some Concerns

BMI = body mass index; NR = not reported.

**Table 3:** Intervention characteristics of randomized controlled trials included in the systematic review examining effect of nutrition and physical activity interventions provided by nutrition and exercise practitioners for the general population.

Trial Name (If Applicable), Study, Author, Year	Nutrition Practitioner	PA Practitioner	Intervention Duration (Months)	Number of Contacts	In-Person, Remote, Blended	Group, Individual, Blended	Diet (Caloric Restriction, Macronutrient Change, Dietary Pattern, Unspecified, Individual)	PA Time (Minutes/Week) and Type (Aerobic, Resistance)	Outcomes Reported
**40-Something Trial** Hollis et al. 2015 [46] Williams et al. 2014 [79] Williams et al. 2019 [80]	Dietitian or international equivalent	Exercise practitioner	12	5	Exclusively In-person	Exclusively Individual	Caloric Restriction, Individualized	150–250, NR	PA F&V Intake WC QoL
Beleigoli et al. 2020 [35]	Dietitian or international equivalent	Dietitian or international equivalent	6	Unclear	Exclusively Remote	Exclusively Individual	NR, Individualized	NR, NR	PA F&V % Weight Loss
Colleran et al. 2012, 2012b [38,39]	Dietitian or international equivalent	Dietitian or international equivalent	4	32	Blended	Exclusively Individual	Caloric Restriction, Dietary Pattern	NR, Both	F&V WC % Weight Loss
**Finnish DPS Trial** Lindstrom et al. 2013 [54] Ruusunen et al. 2012 [72]	Dietitian or international equivalent	Dietitian or international equivalent, Exercise practitioner (description varied between articles)	48	19	Blended	Blended	Caloric Restriction, Macronutrient change, Dietary Pattern, Individualized	240, Both	PA % Weight Loss
Forsyth et al. 2015 [43]	Dietitian or international equivalent	Dietitian or international equivalent	3	4	Exclusively In-person	Exclusively Individual	NR, Individualized	NR, NR	F&V
**GHSH Trial** Fjeldsoe et al. 2016 [42] Fjeldsoe et al. 2019 [41]	Health coach	Health coach	6	2	Exclusively Remote	Exclusively Individual	NR, Individualized	NR, NR	PA F&V WC
Johnson et al. 2019 [50]	Dietitian or international equivalent	Exercise practitioner	3	24	Exclusively In-person	Exclusively Individual	NR, Individualized	150, NR	% Weight Loss
Kennedy et al. 2015 [51]	Dietitian or international equivalent	Dietitian or international equivalent	12	12	Exclusively In-person	Exclusively Group	Unspecified	210, Aerobic	F&V QoL
**LEVA in Real Life Trial** Husenovic et al. 2016 [47] Husenovic et al. 2018 [48]	Dietitian or international equivalent	Dietitian or international equivalent	3	16	Blended	Exclusively Individual	Caloric Restriction, Macronutrient change, Dietary Pattern	NR, NR	WC % Weight Loss
Maddison et al. 2019 [56]	Dietitian or international equivalent	Exercise practitioner	4	12 to 24	Exclusively In-person	Exclusively group	NR	120–150, Both	PA WC
Maruyama et al. 2010 [57]	Dietitian or international equivalent	Exercise practitioner	4	4	Blended	Exclusively Individual	Dietary Pattern, Individualized	NR, NR	WC
**MEDIM Trial** Siddiqui et al. 2017 [73] Siddiqui et al. 2018 [74]	Health coach	Health coach	4	7	Exclusively In-Person	Exclusively group	Dietary Pattern	10,000 steps/day Aerobic	PA WC % Weight Loss
Miller et al. 2015 [59]	Dietitian or international equivalent	Dietitian or international equivalent	4	16	Exclusively In-person	Exclusively Group	Caloric Restriction, Macronutrient change	150, Aerobic	F&V % Weight Loss Adverse events
Neale et al. 2017 [60]	Dietitian or international equivalent	Dietitian or international equivalent OR Exercise practitioner if requested	12	NR	Nutrition: Blended PA: Blended	Nutrition: Exclusively Individual PA: Exclusively Individual	Dietary Pattern, Individualized	NR, NR	PA F&V QoL
**NEW Trial** Abbenhardt et al. 2013 [33] Campbell et al. 2012 [37] Duggan et al. 2016 [40] Foster-Schubert et al. 2012 [44] Imayama et al. 2011 [49] Mason et al. 2011 [58]	Dietitian or international equivalent	Exercise practitioner	12	62	Exclusively In-person	Exclusively Individual	Caloric Restriction, Macronutrient change	225, Aerobic	PA WC % Weight Loss QoL Adverse events
Nicklas et al. 2014 [61]	Dietitian or international equivalent	Dietitian or international equivalent	12	18	Exclusively Remote	Exclusively Individual	Macronutrient change	≤150, Both	PA
Pablos et al. 2017 [62]	Dietitian or international equivalent	Exercise practitioner	8	144	Exclusively In-person	Blended	Caloric Restriction, Macronutrient change, Individualized	140–180, Both	WC
Perri et al. 2020 [65]	Health coach	Health coach	6	18	Exclusively Remote	Individual or Group	Dietary Pattern	210, NR	% Weight Loss
**RAINBOW Trial** Ma et al. 2019 [55] Rosas et al. 2021 [68]	Health coach	Health coach	12	15	Blended	Exclusively Individual	Caloric Restriction	150, NR	PA F&V % Weight Loss
Rich-Edwards et al. 2019 [66]	Dietitian or international equivalent	Dietitian or international equivalent	9	Unclear	Exclusively Remote	Exclusively Individual	Dietary Pattern, Individualized	NR, NR	PA
Rollo et al. 2020 [67]	Dietitian or international equivalent	Exercise practitioner	6	6	Exclusively Remote	Exclusively Individual	NR	NR, Both	PA WC % Weight Loss QoL
Rosas et al. 2020 [69]	Health coach	Health coach	12	22	Exclusively In-person	Blended	Caloric Restriction, Macronutrient change, Dietary Pattern	150, NR	PA F&V WC % Weight Loss QoL
Roumen et al. 2011 [70]	Dietitian or international equivalent	Exercise practitioner	48	16	Exclusively In-person	Exclusively Individual	Caloric Restriction, Macronutrient change, Dietary Pattern, Individualized	150, Both	WC
Rubinstein et al. 2016 [71]	Dietitian or international equivalent	Dietitian or international equivalent	12	12	Exclusively Remote	Exclusively Individual	Macronutrient change, Dietary Pattern, Individualized	NR, NR	PA F&V WC
**Shape Trial** Bennett et al. 2013 [36] Krishnan et al. 2019 [52]	Dietitian or international equivalent	Dietitian or international equivalent	12	12	Exclusively Remote	Exclusively Individual	Caloric Restriction	NR, NR	WC QoL
Thomas et al. 2019 [75]	Dietitian or international equivalent	Exercise practitioner	18	42	Exclusively In-person	Exclusively Group	Caloric Restriction, Macronutrient change	200, NR	% Weight Loss
Toji et al. 2012 [76]	Dietitian or international equivalent	Dietitian or international equivalent, Health fitness programmer	6	7	Exclusively In-person	Blended	Caloric Restriction, Individualized	NR, Both	WC
**TXT2BFiT Trial** Allman-Farinelli et al. 2016 [34] Partridge et al. 2015 [63] Partridge et al. 2016 [64]	Dietitian or international equivalent	Dietitian or international equivalent	9	7	Exclusively Remote	Exclusively Individual	Dietary Pattern	NR, NR	PA F&V
Viester et al. 2018 [77]	Health coach	Health coach	6	2 to 4	Blended	Exclusively Individual	NR Individualized	NR, Resistance	PA F&V WC
Weinhold et al. 2015 [78]	Dietitian or international equivalent	Dietitian or international equivalent	4	12	Exclusively In-person	Blended	Caloric Restriction, Macronutrient change, Individualized	≤150, NR	PA WC % Weight Loss
**WOMAN Trial** Gabriel et al. 2011 [45] Kuller et al. 2012 [53]	Dietitian or international equivalent	Exercise practitioner	36	64	Exclusively In-person	Blended	Caloric Restriction, Dietary Pattern	NR, NR	PA WC % Weight Loss

F&V = fruit and vegetable, NR = not reported, PA = physical activity, QoL = quality of life, WC = waist circumference.

**Table 4:** Summary of findings describing effect of nutrition and physical activity interventions provided by nutrition and exercise practitioners for the general population.

Outcome Number of Participants (Studies)	Anticipated Absolute Effects (95% Confidence Interval (CI))	Risk of Bias	Inconsistency	Indirectness	Imprecision	Other	Evidence Certainty	What Happens
Physical activity amount Participants: 3339 (13 randomized controlled trials (RCTs))	Standardized Mean Difference (SMD) 0.25 SD higher (0.08 higher to 0.42 higher)	◼ ^a^	◼	◻ ^b^	◻	◻	⨁⨁◯◯ LOW	In adults who are healthy or have cardiometabolic risk factors, nutrition and physical activity interventions from nutrition and exercise practitioners may increase physical activity amount.
Fruit Participants: 1839 (9 RCTs)	SMD 0.38 SD higher (0.12 higher to 0.63 higher)	◼	◼	◻	◻	◻	⨁⨁◯◯ LOW	In adults who are healthy, nutrition and physical activity interventions from nutrition and exercise practitioners may increase fruit intake, but results are more heterogeneous for adults with cardiometabolic risk factors.
Vegetable intake Participants: 1839 (9 RCTs)	SMD 0.14 SD higher (0.05 higher to 0.23 higher)	◼	◻	◻	◻	◻	⨁⨁⨁◯ MODERATE	In adults who are healthy, nutrition and physical activity interventions from nutrition and exercise practitioners likely increases vegetable intake slightly, but results were not significant for adults with cardiometabolic risk factors.
Waist circumference (cm) Participants: 2776 (18 RCTs)	Mean Difference (MD) 2.16 cm lower (2.96 lower to 1.36 lower)	◼	◻	◻	◻	◼ ^c^	⨁⨁⨁⨁ HIGH	In adults who have cardiometabolic risk factors, nutrition and physical activity interventions from nutrition and exercise practitioners reduce waist circumference compared to controls across a wide range of interventions, but results were not significant in studies targeting healthy adults.
Achieving 5% Weight Loss For participants with overweight or obesity Participants: 1112 (8 RCTs)	Relative Risk 2.37 (1.76 to 3.19)	◼	◻	◻	◻	◼ ^d^	⨁⨁⨁⨁ HIGH	In adults with overweight or obesity but no diagnosed disease, nutrition and physical activity interventions from nutrition and exercise practitioners improved likelihood of achieving 5% weight loss compared to controls.
Percent weight loss (continuous) For participants with overweight or obesity Participants: 1030 (7 RCTs)	MD 2.37% lower (5.51 lower to 0.77 higher)	◼	◼	◻	◻	◻	⨁⨁◯◯ LOW	In adults with overweight or obesity but no diagnosed disease, nutrition and physical activity interventions from nutrition and exercise practitioners, there was no significant reduction in percent weight loss as a continuous variable compared to controls and heterogeneity was high.
Quality of Life Participants: 295 (3 RCTs)	MD 3.91 higher (0.21 lower to 8.03 higher)	◼	◼	◻	◼	◻	⨁◯◯◯ VERY LOW	In adults who are healthy or have cardiometabolic risk factors, the evidence is very uncertain about the effect of nutrition and physical activity interventions provided by nutrition and exercise practitioners on physical and mental quality of life but suggests little-to-no effect.
Adverse events Participants: (3 RCTs)	not pooled	◼	◻	◻	◼	◻	⨁⨁◯◯ LOW	Nutrition and physical activity interventions provided by nutrition and exercise practitioners may result in little to no difference in adverse events, though postmenopausal women receiving the intervention had reduced bone mineral density compared to the control group in one study.

^a^ ◼ Indicates certainty of evidence was marked down for risk of bias, inconsistency, indirectness and imprecision or marked up or down for other reasons. ^b^ ◻ indicates certainty of evidence was not marked up or down for the respective reason. ^c^ Dose-Response effect demonstrated ^d^ Large effect size.

## Data Availability

No new data were created or analyzed in this study. Data sharing is not applicable to this article.

## Supplementary Materials

The following supporting information can be downloaded at: https://www.mdpi.com/article/10.3390/nu14091729/s1. Figure S1. Publication Bias of Studies Included in the Systematic Review Examining the Effects of Nutrition and Physical Activity Interventions Provided by Qualified Practitioners; Table S1. Full search strategy for literature search of databases for the systematic review examining the effect of nutrition and physical activity interventions.
