# Supplementary material for: Impact of Nutrition and Physical Activity Interventions Provided by Nutrition and Exercise Practitioners for the Adult General Population: A Systematic Review and Meta-Analysis
Source: Nutrients. 2022 Apr 21;14(9):1729. doi: 10.3390/nu14091729 (PMC9103154; doi:10.3390/nu14091729)
Supplement: Supplementary file 1 [file nutrients-14-01729-s001.zip › Supplementary Material/Supplementary Figure 1. Publication Bias.pdf]

Supplementary Figure S1. Publication Bias of Studies Included in the Systematic Review Examining the Effects of Nutrition and Physical Activity Interventions Provided by Qualified Practitioners

A. Funnel Plot for Publication Bias for the Outcome of Physical Activity

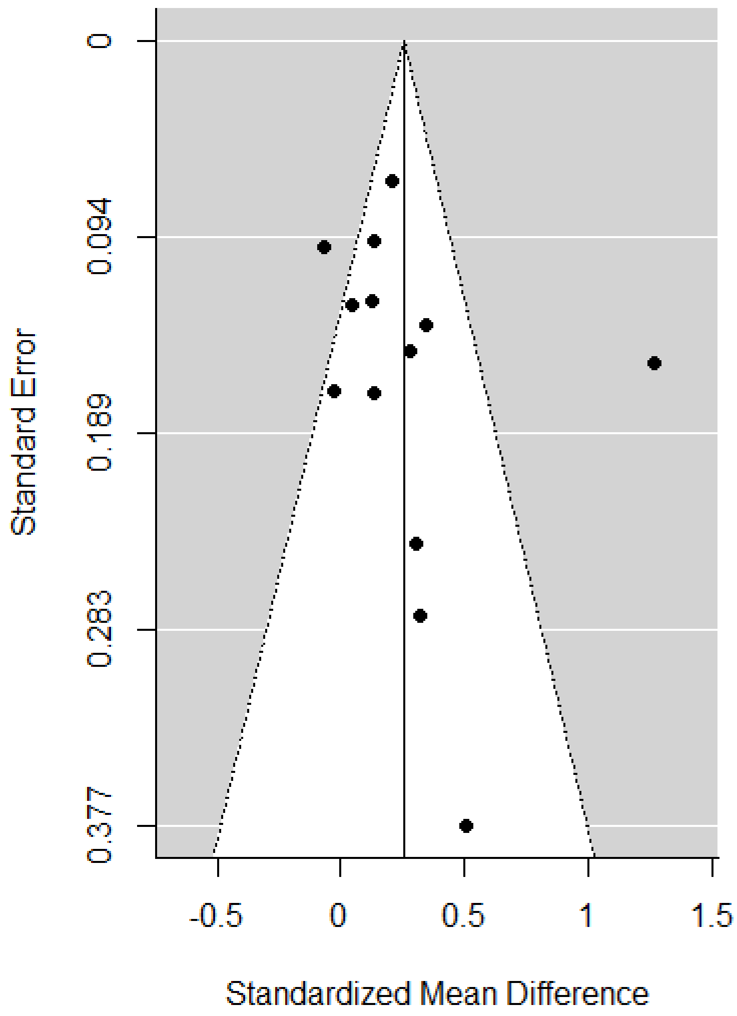

Egger’s test: z=0.9379, p=0.3483

B. Funnel Plot for Publication Bias for the Outcome of Fruit Intake

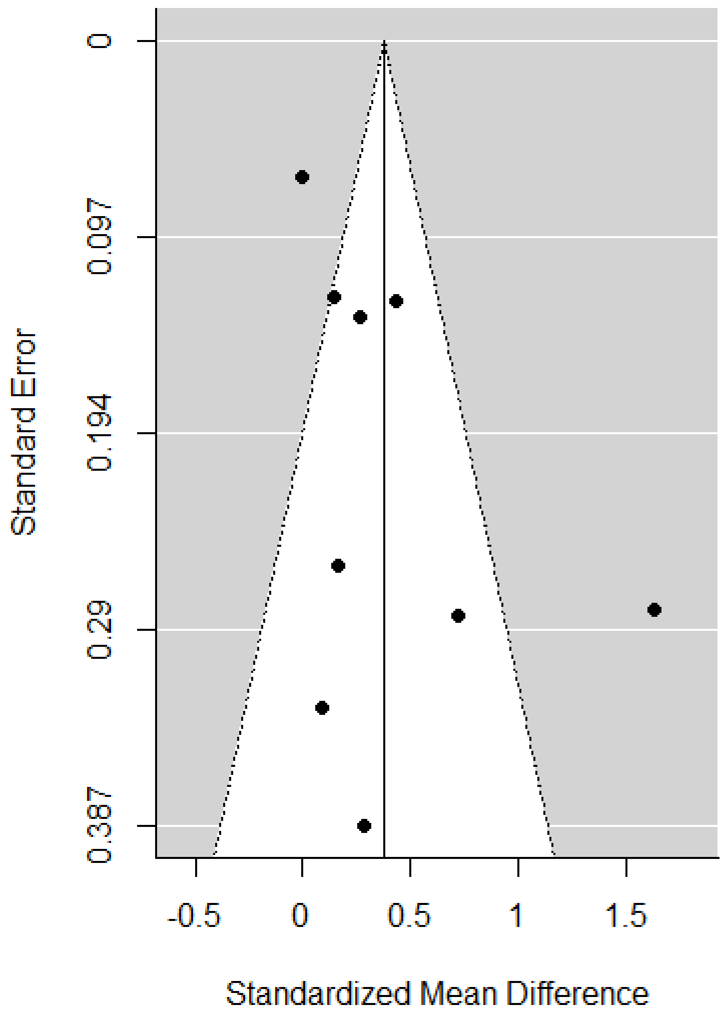

Egger's test z=1.5603, p=0.1187

C. Funnel Plot for Publication for the Outcome of Waist Circumference.

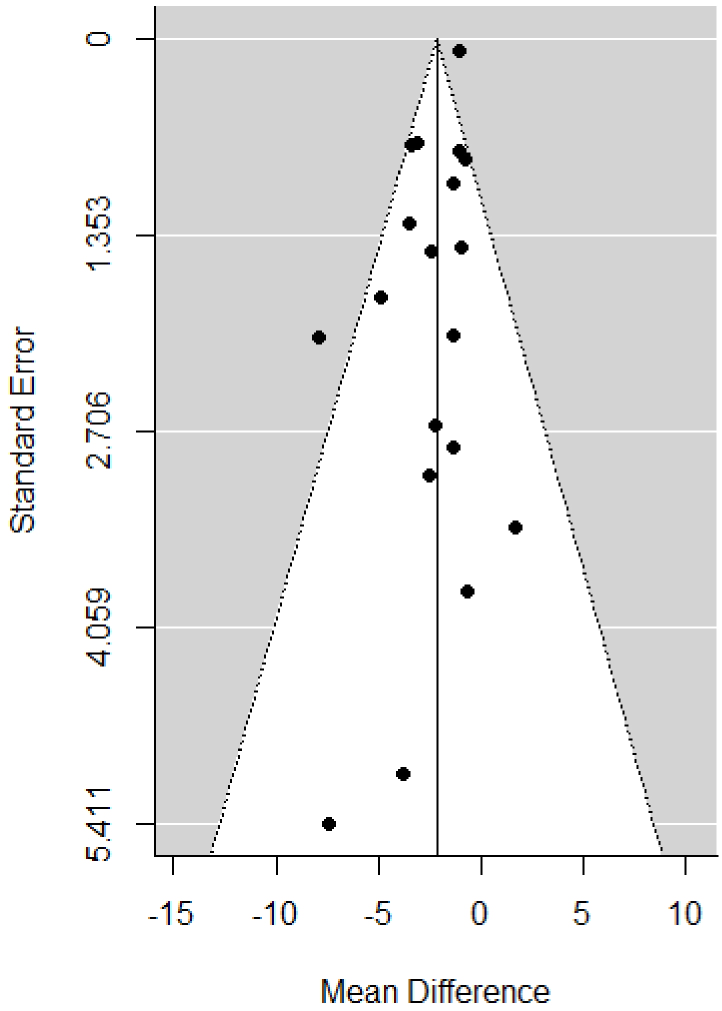

Eggers test: z=-1.1695, p=0.2422

D. Funnel Plot for Publication Bias for the Outcome of 5% Weight Loss

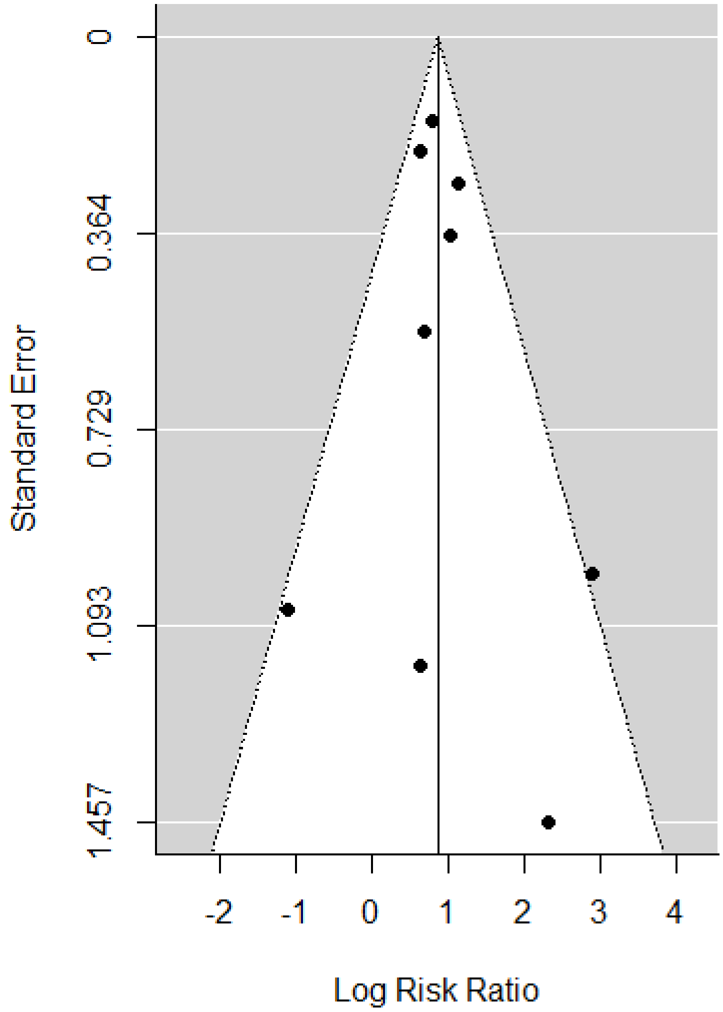

Eggers test: z=0.5572, p=0.5774

E. Funnel Plot for Publication Bias for the Outcome of Glucose levels

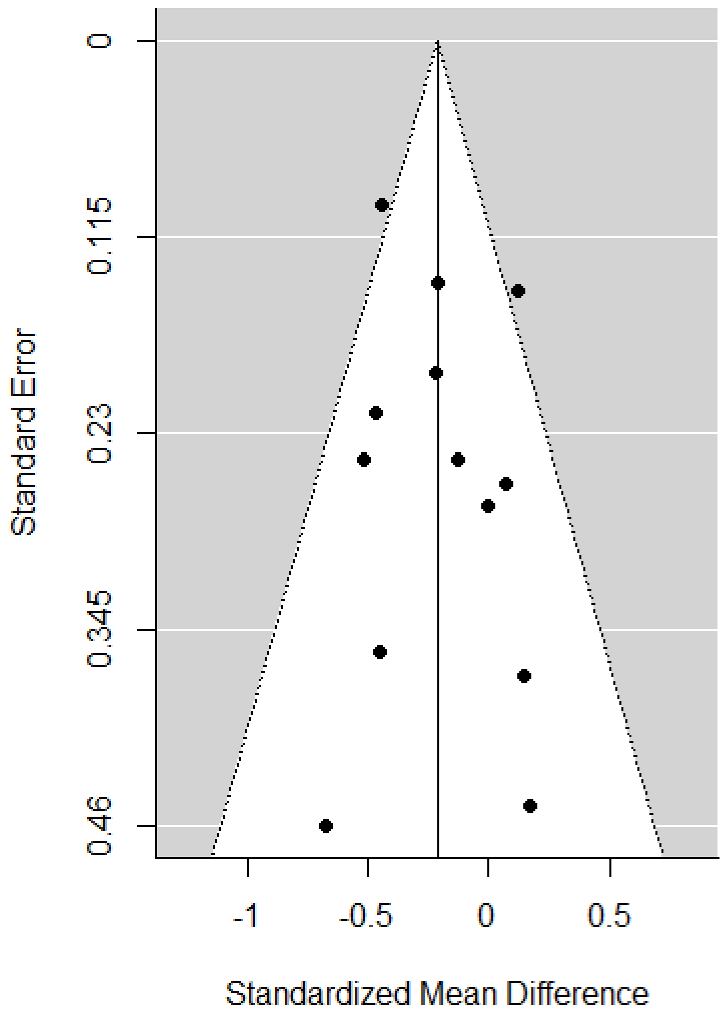

Egger's Test z = 0.4213, p = 0.6735

F. Funnel Plot for Publication Bias for the Outcome of Physical QoL

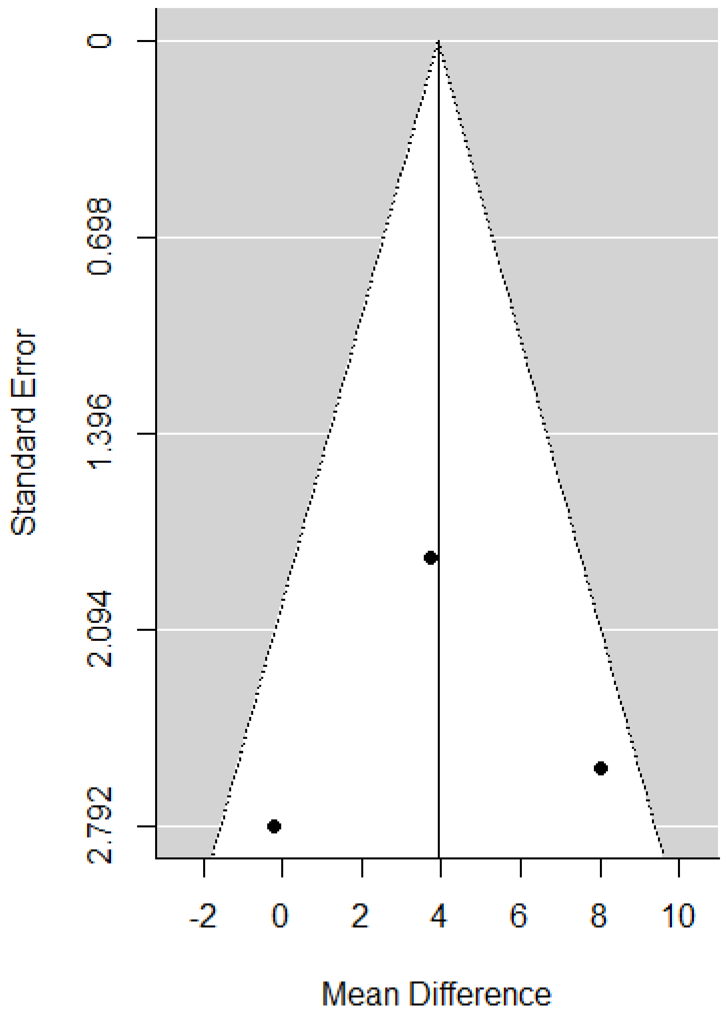

Eggers test: z=-0.1629, p=0.8706
