# Supplementary material for: Impact of Nutrition and Physical Activity Interventions Provided by Nutrition and Exercise Practitioners for the Adult General Population: A Systematic Review and Meta-Analysis
Source: Nutrients. 2022 Apr 21;14(9):1729. doi: 10.3390/nu14091729 (PMC9103154; doi:10.3390/nu14091729)
Supplement: Supplementary file 1 [file nutrients-14-01729-s001.zip › Supplementary Material/Supplementary Table 1.pdf]

Table S1. Full search strategy for literature search of databases for the systematic review examining the effect of nutrition and physical activity interventions.

**Search Date**	**Database**	**Results**
April-2-2021	Medline Complete (Ebsco)	8,787
April-2-2021	Medline Complete (Ebsco)	496
April-2-2021	CINAHL (Ebsco)	4755
March-28-2021	Cochrane Database of Systematic Reviews (Ebsco)	9
April-2-2021	Cochrane Central Database of Controlled Trials (Ebsco)	5538
April-2-2021	SportDiscus (Ebsco)	989
	Total before duplicate removal	20,574
	Duplicates	9369
	Total (Sent for Title/abstract screening)	11205

MEDLINE

#	Query [Medline Complete, Ebsco, April 2, 2021]	Results	Limiters/Expanders
1	MH "Diet+"	290,364	Search modes - Boolean/Phrase
2	TI (diet or diets or dietary or nutrition*) OR AB (diet or diets)	506,928	Search modes - Boolean/Phrase
3	MH "Eating"	53,870	Search modes - Boolean/Phrase
4	TI eating	27,057	Search modes - Boolean/Phrase
5	AB (eating habit or eating habits) OR AB (dietary intake OR dietary habit*)	63,343	Search modes - Boolean/Phrase
6	AB ((healthy OR unhealthy or nutritional) N2 (eating or meal or meals))	9,593	Search modes - Boolean/Phrase
7	S1 OR S2 OR S3 OR S4 OR S5 OR S6	703,679	Search modes - Boolean/Phrase
8	MH "Exercise+"	205,240	Search modes - Boolean/Phrase
9	TI (exercise or exercising or exercised or physical activity or physical activities OR aerobic activity or aerobic activities OR ((aerobic* or fitness or pilates or yoga) N2 (class or classes))	270,818	Search modes - Boolean/Phrase
10	AB (physical activity or physical activities or physical fitness or fitness routine or fitness class or fitness classes or aerobic exercis*) OR AB ((regular or daily or routine or weekly or week or weeks) N2 (exercis* or gym))	150,235	Search modes - Boolean/Phrase
11	TI ((regular or daily or routine or weekly or week or weeks) N2 gym)	2	Search modes - Boolean/Phrase
12	AB (aerobic activity or aerobic activities OR ((aerobic* or fitness or pilates or yoga) N2 (class or classes))	4,919	Search modes - Boolean/Phrase
13	S8 OR S9 OR S10 OR S11 OR S12	384,203	Search modes - Boolean/Phrase
14	MH "Muscle Strength+"	35,931	Search modes - Boolean/Phrase

15	TI (anthropomorphic* N2 (measur* OR test# or testing or tested)) OR AB (anthropomorphic* N2 (measur* OR test# or testing or tested))	704	Search modes - Boolean/Phrase
16	S14 OR S15	36,633	Search modes - Boolean/Phrase
17	(MH "Cognitive Behavioral Therapy+") OR (MH "Behavior Therapy+") OR (MH "Directive Counseling+") OR (MH "Counseling")	117,018	Search modes - Boolean/Phrase
18	(TI (counseling or counselling or counselor* or counsellor* or motivational interview* or directive therapy or ((behavior* or behaviour*) N2 (intervention or interventions OR therapy or therapies)))) OR (AB (counseling or counselling OR motivational interview* or directive therapy or ((behavior* or behaviour*) N2 (therapy or therapies))))	129,118	Search modes - Boolean/Phrase
19	S17 OR S18	205,895	Search modes - Boolean/Phrase
20	MH "Overweight" OR MH "Obesity+"	230,985	Search modes - Boolean/Phrase
21	TI (overweight or obese or obesity) OR AB (overweight or obese or obesity)	331,309	Search modes - Boolean/Phrase
22	S20 OR S21	381,450	Search modes - Boolean/Phrase
23	(MH "Randomized Controlled Trials as Topic+")	145,086	Search modes - Boolean/Phrase
24	(ZT "randomized controlled trial")	524,962	Search modes - Boolean/Phrase
25	(TI (randomised or randomized OR trial)) OR (AB (randomized or randomised or randomly))	1,061,371	Search modes - Boolean/Phrase
26	(ZT "controlled clinical trial")	94,070	Search modes - Boolean/Phrase
27	TI (placebo*) OR AB (placebo*)	223,105	Search modes - Boolean/Phrase
28	(MH "Randomized Controlled Trial")	524,962	Search modes - Boolean/Phrase

29	S23 OR S24 OR S25 OR S26 OR S27 OR S28	1,364,479	Search modes - Boolean/Phrase
30	MH ("Animals+") NOT MH ("humans")	4,800,917	Search modes - Boolean/Phrase
31	((MH "Child+") OR (MH "Infant+") OR (MH "Adolescent")) NOT (((MH "Adult+") OR (MH "Young Adult") OR (MH "Middle Aged")))	1,919,039	Search modes - Boolean/Phrase
32	TI (adolescent* or teen or teens or teenager* OR child or childs OR children* or pediatric* or paediatric* OR nursery or newborn* or new born* or infant or infants)	1,192,203	Search modes - Boolean/Phrase
33	AB (adolescent* or teen or teens or teenager* OR child or childs OR children* or pediatric* or paediatric* OR nursery or newborn* or new born* or infant or infants)	1,531,008	Search modes - Boolean/Phrase
34	TI (schoolage* or school age* OR school based or school based or kindergar* or preschool* or pre-school* or elementary school* or junior high* or highschool*) or TI (K-12 N2 (student* or school* or education*))	37,792	Search modes - Boolean/Phrase
35	AB (schoolage* or school age* OR school based or school based or kindergar* or preschool* or pre-school* or elementary school* or junior high* or highschool*) OR AB (K-12 N2 (student* or school* or education*))	86,305	Search modes - Boolean/Phrase
36	MH ("Schools, Nursery")	1,481	Search modes - Boolean/Phrase
37	S31 OR S32 OR S34 OR S35 OR S36	2,294,843	Search modes - Boolean/Phrase
38	(AB ((systematic review or scoping review or meta-analy* or metaanyl*)) OR TI (((systematic or scoping or review) N2 (review or reviews)) or scoping review or meta-analy* or metaanyl*)) OR MW ((systematic review or scoping review or meta-analy* or metaanyl*))) OR SO (cochrane OR Systematic review*) OR (AB	459,697	Limiters - English Language

	"risk of bias" or quality assessment or data extraction or Pubmed or Medline OR "web of science" or Embase OR CINAHL) OR (ZT "systematic review") OR (ZT "Meta-analysis") OR TI meta-synthesis OR AB meta-synthesis		
39	S7 AND (S13 OR S16)	50,648	Search modes - Boolean/Phrase
40	(S13 OR S16) AND S19	10,900	Search modes - Boolean/Phrase
41	S22 AND S19	8,979	Search modes - Boolean/Phrase
42	S39 OR S40 OR S41	63,926	Search modes - Boolean/Phrase
43	S39 OR S40 OR S41	60,374	Narrow by Language: - english
44	S39 OR S40 OR S41	39,095	Limiters - Date of Publication: 20100101-20211231
45	S44 AND S29	10,798	Limiters - Date of Publication: 20100101-20211231
46	S45 NOT S30	10,493	Limiters - Date of Publication: 20100101-20211231
47	S46 NOT S37	8,787	Search modes - Boolean/Phrase
48	S38 AND S43	3,355	Search modes - Boolean/Phrase
49	S48 NOT S37	2,737	Search modes - Boolean/Phrase
50	S48 NOT S37	496	Limiters - Date of Publication: 20200101-20211231

CINAHL

#	**Query [CINAHL, Ebsco Host, April 2, 2021]**	**Results**	**Last Run Via**
1	(MH "Diet+") OR (MH "Food Intake+")	126,163	Interface - EBSCOhost Research Databases
2	TI (diet or diets or dietary or nutrition*) OR AB (diet or diets)	140,498	Interface - EBSCOhost Research Databases
3	MH "Eating"	6,804	Interface - EBSCOhost Research Databases
4	TI eating	18,473	Interface - EBSCOhost Research Databases
5	AB (eating habit or eating habits) OR AB (dietary intake OR dietary habit*)	21,649	Interface - EBSCOhost Research Databases
6	AB ((healthy OR unhealthy or nutritional) N2 (eating or meal or meals))	6,787	Interface - EBSCOhost Research Databases
7	S1 OR S2 OR S3 OR S4 OR S5 OR S6	237,193	Interface - EBSCOhost Research Databases
8	(MH "Exercise+") OR (MH "Physical Fitness+")	130,435	Interface - EBSCOhost Research Databases
9	TI (exercise or exercising or exercised or physical activity or physical activities OR aerobic activity or aerobic activities OR ((aerobic* or fitness or pilates or yoga) N2 (class or classes))	133,285	Interface - EBSCOhost Research Databases
10	AB (physical activity or physical activities or physical fitness or fitness routine or fitness class or fitness classes or aerobic exercis*) OR AB ((regular or daily or routine or weekly or week or weeks) N2 (exercis* or gym))	70,332	Interface - EBSCOhost Research Databases
11	TI ((regular or daily or routine or weekly or week or weeks) N2 gym)	6	Interface - EBSCOhost Research Databases

12	AB (aerobic activity or aerobic activities OR ((aerobic* or fitness or pilates or yoga) N2 (class or classes))	1,811	Interface - EBSCOhost Research Databases
13	S8 OR S9 OR S10 OR S11 OR S12	220,224	Interface - EBSCOhost Research Databases
14	MH "Muscle Strength+"	26,077	Interface - EBSCOhost Research Databases
15	TI anthropomorphic* measur* OR AB anthropomorphic* measur*	139	Interface - EBSCOhost Research Databases
16	S14 OR S15	26,211	Interface - EBSCOhost Research Databases
17	((MH "Cognitive Therapy+") OR (MH "Behavior Therapy+")) OR ((MH "Motivational Interviewing") OR (MH "Peer Counseling") OR (MH "Anticipatory Guidance")) OR (MH "Nutritional Counseling")	43,414	Interface - EBSCOhost Research Databases
18	(TI (counseling or counselling or counselor* or counsellor* or motivational interview* or directive therapy or ((behavior* or behaviour*) N2 (intervention or interventions OR therapy or therapies)))) OR (AB (counseling or counselling OR motivational interview* or directive therapy or ((behavior* or behaviour*) N2 (therapy or therapies))))	68,980	Interface - EBSCOhost Research Databases
19	S17 OR S18	99,227	Interface - EBSCOhost Research Databases
20	MH "Obesity+"	103,807	Interface - EBSCOhost Research Databases
21	TI (overweight or obese or obesity) OR AB (overweight or obese or obesity)	113,095	Interface - EBSCOhost Research Databases
22	S20 OR S21	144,974	Interface - EBSCOhost Research Databases

23	(MH "Randomized Controlled Trials as Topic+")	2,040	Interface - EBSCOhost Research Databases
24	(ZT "randomized controlled trial")	128,380	Interface - EBSCOhost Research Databases
25	(TI (randomised or randomized)) OR (AB (randomized or randomised or randomly))	319,894	Interface - EBSCOhost Research Databases
26	(ZT "controlled clinical trial")	54	Interface - EBSCOhost Research Databases
27	TI (placebo*) OR AB (placebo*)	64,955	Interface - EBSCOhost Research Databases
28	(MH "Randomized Controlled Trial")	1,528	Interface - EBSCOhost Research Databases
29	S23 OR S24 OR S25 OR S26 OR S27 OR S28	362,532	Interface - EBSCOhost Research Databases
30	MH ("Animals+") NOT MH ("humans")	96,658	Interface - EBSCOhost Research Databases
31	S7 AND (S13 OR S16)	32,898	Interface - EBSCOhost Research Databases
32	(S13 OR S16) AND S19	5,855	Interface - EBSCOhost Research Databases
33	S22 AND S19	4,260	Interface - EBSCOhost Research Databases
34	S31 OR S32 OR S33	39,502	Interface - EBSCOhost Research Databases
35	S29 AND S34	6,896	Interface - EBSCOhost Research Databases
36	S35 NOT S30	6,882	Interface - EBSCOhost Research Databases
37	((MH "Child+") OR (MH "Infant+") OR (MH "Adolescence+")) NOT ((MH "Adult+") OR (MH "Middle Age") OR (MH "Aged+") OR (MH "Young Adult"))	624,210	Interface - EBSCOhost Research Databases

38	TI (adolescent* or teen or teens or teenager* OR child or childs OR children* or pediatric* or paediatric* OR nursery or newborn* or new born* or infant or infants)	459,925	Interface - EBSCOhost Research Databases
39	AB (adolescent* or teen or teens or teenager* OR child or childs OR children* or pediatric* or paediatric* OR nursery or newborn* or new born* or infant or infants)	517,756	Interface - EBSCOhost Research Databases
40	TI (schoolage* or school age* OR school based or school based or kindergar* or preschool* or pre-school* or elementary school* or junior high* or highschool*) or TI (K-12 N2 (student* or school* or education*))	20,653	Interface - EBSCOhost Research Databases
41	AB (schoolage* or school age* OR school based or school based or kindergar* or preschool* or pre-school* or elementary school* or junior high* or highschool*) OR AB (K-12 N2 (student* or school* or education*))	41,203	Interface - EBSCOhost Research Databases
42	MH ("Schools, Nursery")	1,373	Interface - EBSCOhost Research Databases
43	S37 OR S38 OR S40 OR S41 OR S42	788,980	Interface - EBSCOhost Research Databases
44	S36 NOT S43	5,805	Interface - EBSCOhost Research Databases
45	S36 NOT S43	4,637	Interface - EBSCOhost Research Databases
46	S36 NOT S43	4,553	Interface - EBSCOhost Research Databases
47	(AB ((systematic review or scoping review or meta-analy* or metaanyl*)) OR TI (((systematic or scoping or review) N2 (review or reviews)) or scoping review or meta-analy* or metaanyl*)) OR MW ((systematic review or scoping review or meta-analy* or metaanyl*))) OR SO (cochrane OR Systematic review*) OR (AB	240,361	Interface - EBSCOhost Research Databases

	"risk of bias" or quality assessment or data extraction or Pubmed or Medline OR "web of science" or Embase OR CINAHL) OR (MM "systematic review") OR (MM "Meta-analysis") OR TI meta-synthesis OR AB meta-synthesis		
48	S34 AND S47	2,089	Interface - EBSCOhost Research Databases
49	S34 AND S47	1,784	Interface - EBSCOhost Research Databases
50	S34 AND S47	280	Interface - EBSCOhost Research Databases
51	S46 OR S50	4,755	Interface - EBSCOhost Research Databases

Sport Discus

#	Query [SPORT-DISCUS] April 2, 2021	Results	Limiters/Expanders
1	DE "PHYSICAL fitness & nutrition"	188	Search modes - Boolean/Phrase
2	DE "DIET" OR DE "FASTING" OR DE "FOOD portions" OR DE "HIGH-calcium diet" OR DE "HIGH-carbohydrate diet" OR DE "HIGH-fat diet" OR DE "HIGH-fiber diet" OR DE "MEDITERRANEAN diet" OR DE "REDUCING diets" OR DE "TYRAMINE-free diet" OR DE "VEGETARIANISM" OR DE "DIET therapy" OR DE "CASEIN-free diet" OR DE "COMMUNICATION in diet therapy" OR DE "COMPLEX carbohydrate diet" OR DE "ELEMENTAL diet" OR DE "FOOD exchange lists" OR DE "GLUTEN-free diet" OR DE "HIGH-calcium diet" OR DE "HIGH-calorie diet" OR DE "HIGH-carbohydrate diet" OR DE "HIGH-iron diet" OR DE "HIGH-lysine diet" OR DE "HIGH-omega-3 fatty acid diet" OR DE "HIGH-potassium diet" OR DE "HIGH-protein diet" OR DE "HIGH-tryptophan diet" OR DE "LIQUID diet" OR DE "LOW-potassium diet" OR DE "RAW food diet" OR DE "TYRAMINE-free diet" OR DE "WHEAT-free diet" OR DE "YEAST-free diet" OR ((DE "FOOD habits") OR (DE "FOOD portions")) OR (DE "FOOD consumption")	24,723	Search modes - Boolean/Phrase
3	TI eating OR TI (diet or diets or dietary or nutrition*) OR AB (diet or diets) OR KW (diet or diets or dietary or nutrition*)	53,351	Search modes - Boolean/Phrase
4	AB (eating habit or eating habits) OR AB (dietary intake OR dietary habit*) OR TI (food habit$) OR TI (food consumption) OR KW (food habit$) OR KW (food consumption)	7,753	Search modes - Boolean/Phrase
5	AB ((healthy OR unhealthy or nutritional) N2 (eating or meal or meals)) OR KW ((healthy OR	2,665	Search modes - Boolean/Phrase

	unhealthy or nutritional) N2 (eating or meal or meals))		
6	S2 OR S3 OR S4 OR S5	65,187	Search modes - Boolean/Phrase
7	(DE "FITNESS walking") AND (DE "PHYSICAL fitness" OR DE "ANAEROBIC exercises" OR DE "CARDIOPULMONARY fitness" OR DE "CARDIOVASCULAR fitness" OR DE "CIRCUIT training" OR DE "COMPOUND exercises" OR DE "EXERCISE tolerance" OR DE "ISOLATION exercises" OR DE "LIANGONG" OR DE "MUSCLE strength" OR DE "PERIODIZATION training" OR DE "PHYSICAL fitness for men" OR DE "PHYSICAL fitness for older people" OR DE "PHYSICAL fitness for people with disabilities" OR DE "PHYSICAL fitness for women" OR DE "SPORT for all" OR DE "PHYSICAL fitness -- Research" OR DE "PHYSICAL fitness centers" OR DE "HOME gyms" OR DE "WEIGHT training facilities")	115	Search modes - Boolean/Phrase
8	TI (exercise or exercising or exercised or physical activity or physical activities OR aerobic activity or aerobic activities) OR TI ((aerobic* or fitness or pilates or yoga) N2 (class or classes))	92,081	Search modes - Boolean/Phrase
9	AB (physical activity or physical activities or physical fitness or fitness routine or fitness class or fitness classes or aerobic exercis*) OR AB ((regular or daily or routine or weekly or week or weeks) N2 (exercis* or gym)) OR KW ((regular or daily or routine or weekly or week or weeks) N2 (exercis* or gym)) OR KW (physical activity or physical activities or physcial fitness or fitness routine or fitness class or fitness classes or aerobic exercis*)	64,792	Search modes - Boolean/Phrase
10	TI ((regular or daily or routine or weekly or week or weeks) N2 gym) OR AB (aerobic	2,873	Search modes - Boolean/Phrase

	activity or aerobic activities OR ((aerobic* or fitness or pilates or yoga) N2 (class or classes))		
11	TI (anthropomorphic N2 (measure* or test or tests or testing)) OR AB (anthropomorphic N2 (measure* or test or tests or testing)) OR KW (anthropomorphic N2 (measure* or test or tests or testing))	63	Search modes - Boolean/Phrase
12	S7 OR S8 OR S9 OR S10 OR S11	131,903	Search modes - Boolean/Phrase
13	(DE "COUNSELING" OR DE "HEALTH counseling" OR DE "LEISURE counseling" OR DE "MENTORING" OR DE "MOTIVATIONAL interviewing") OR (DE "COGNITIVE therapy")	4,647	Search modes - Boolean/Phrase
14	(TI (counseling or counselling or counselor* or counsellor* or motivational interview* or directive therapy or ((behavior* or behaviour*) N2 (intervention or interventions OR therapy or therapies)))) OR (AB (counseling or counselling OR motivational interview* or directive therapy or ((behavior* or behaviour*) N2 (therapy or therapies))))	7,097	Search modes - Boolean/Phrase
15	S13 OR S14	9,794	Search modes - Boolean/Phrase
16	DE "OVERWEIGHT persons" OR DE "OVERWEIGHT men" OR DE "OVERWEIGHT women" OR DE "obesity"	13,922	Search modes - Boolean/Phrase
17	TI (overweight or obese or obesity) OR AB (overweight or obese or obesity)	27,772	Search modes - Boolean/Phrase
18	S16 OR S17	29,920	Search modes - Boolean/Phrase
19	(TI (randomised or randomized)) OR (AB (randomized or randomised or randomly))	48,909	Search modes - Boolean/Phrase
20	TI (controlled N2 trial) OR AB (controlled N2 trial) OR KW (controlled N2 trial)	15,995	Search modes - Boolean/Phrase
21	TI (placebo*) OR AB (placebo*)	12,287	Search modes - Boolean/Phrase

22	S19 OR S20 OR S21	55,426	Search modes - Boolean/Phrase
23	DE "ANIMAL body composition" OR DE "ANIMAL breeding" OR DE "ANIMAL diseases" OR DE "ANIMAL feeds" OR DE "ANIMAL fighting" OR DE "ANIMAL grooming" OR DE "ANIMAL locomotion" OR DE "ANIMAL jumping" OR DE "ANIMAL mechanics" OR DE "ANIMAL locomotion" OR DE "ANIMAL models in food consumption research" OR TI animal# OR AB "animal model#" or KW "animal model#"	5,469	Search modes - Boolean/Phrase
24	(DE "CHILDREN" OR DE "AIDS & children" OR DE "BOYS" OR DE "CHILD acrobats" OR DE "CHILD circus performers" OR DE "CHILD dancers" OR DE "CHILD development" OR DE "DANCE for children" OR DE "DEAFBLIND children" OR DE "GIRLS" OR DE "OUTDOOR recreation for children" OR DE "SCHOOL children" OR DE "SELF-defense for children" OR DE "VIDEO games & children") OR (DE "TEENAGERS" OR DE "OVERWEIGHT teenagers" OR DE "VIDEO games & teenagers") OR (DE "PEDIATRIC nutritionists") OR (DE "PEDIATRICS")	65,707	Search modes - Boolean/Phrase
25	TI (adolescent* or teen or teens or teenager* OR child or childs OR children* or pediatric* or paediatric* OR nursery or newborn* or new born* or infant or infants)	56,872	Search modes - Boolean/Phrase
26	AB (adolescent* or teen or teens or teenager* OR child or childs OR children* or pediatric* or paediatric* OR nursery or newborn* or new born* or infant or infants)	88,118	Search modes - Boolean/Phrase
27	TI (schoolage* or school age* OR school based or school based or kindergar* or preschool* or pre-school* or elementary school* or junior high* or highschool*) or TI (K-12 N2 (student* or school* or education*))	7,125	Search modes - Boolean/Phrase

28	AB (schoolage* or school age* OR school based or school based or kindergar* or preschool* or pre-school* or elementary school* or junior high* or highschool*) OR AB (K-12 N2 (student* or school* or education*))	14,759	Search modes - Boolean/Phrase
29	S24 OR S25 OR S27 OR S28	111,519	Search modes - Boolean/Phrase
30	(AB ((systematic review or scoping review or meta-analy* or metaanyl*)) OR TI (((systematic or scoping or review) N2 (review or reviews)) or scoping review or meta-analy* or metaanyl*)) OR MW ((systematic review or scoping review or meta-analy* or metaanyl*))) OR SO (cochrane OR Systematic review*) OR (AB "risk of bias" or quality assessment or data extraction or Pubmed or Medline OR "web of science" or Embase OR CINAHL) OR TI meta-synthesis OR AB meta-synthesis OR KW ((systematic review or scoping review or meta-analy* or metaanyl*)	20,653	Search modes - Boolean/Phrase
31	S1 OR (S6 AND S12)	8,798	Search modes - Boolean/Phrase
32	S12 AND S15	1,081	Search modes - Boolean/Phrase
33	S18 AND S15	507	Search modes - Boolean/Phrase
34	S31 OR S32 OR S33	9,979	Search modes - Boolean/Phrase
35	(S34 AND S22) NOT (S23 OR S29)	1,374	Search modes - Boolean/Phrase
36	(S34 AND S22) NOT (S23 OR S29)	971	Limiters - Published Date: 20100101-20211231
37	(S34 AND S22) NOT (S23 OR S29)	971	Limiters - Published Date: 20100101-20211231
38	S34 AND S30	261	Limiters - Published Date: 20100101-20211231

39	S34 AND S30	34	Limiters - Published Date: 20200101-20211231
40	S34 AND S30	34	Limiters - Published Date: 20200101-20211231
41	S37 OR S40	989	Search modes - Boolean/Phrase

Cochrane Database of Systematic Reviews

#	**Query Cochrane Database of Systematic Reviews, Ebsco, March 28, 2021]**	**Results**
1	(MM "Diet")	12
2	TI (diet or diets or dietary or nutrition*) OR AB (diet or diets)	430
3	MM "Eating"	6
4	TI eating	6
5	AB (eating habit or eating habits) OR AB (dietary intake OR dietary habit*)	82
6	AB ((healthy OR unhealthy or nutritional) N2 (eating or meal or meals))	19
7	S1 OR S2 OR S3 OR S4 OR S5 OR S6	466
8	(MM "Exercise+") OR (MM "Physical Fitness+")	53
9	TI (exercise or exercising or exercised or physical activity or physical activities OR aerobic activity or aerobic activities) OR TI ((aerobic* or fitness or pilates or yoga) N2 (class or classes))	238
10	AB (physical activity or physical activities or physical fitness or fitness routine or fitness class or fitness classes or aerobic exercis*) OR AB ((regular or daily or routine or weekly or week or weeks) N2 (exercis* or gym))	258
11	TI ((regular or daily or routine or weekly or week or weeks) N2 gym)	3
12	AB (aerobic activity or aerobic activities OR ((aerobic* or fitness or pilates or yoga) N2 (class or classes))	11
13	S8 OR S9 OR S10 OR S11 OR S12	378
14	MM "Muscle Strength+"	110
15	TI anthropomorphic* measur* OR AB anthropomorphic* measur*	0
16	S14 OR S15	0
17	((MM "Cognitive Therapy+") OR (MM "Behavior Therapy+")) OR ((MM "Motivational Interviewing") OR (MM "Peer Counseling") OR (MM "Anticipatory Guidance")) OR (MM "Nutritional Counseling")	1
18	(TI (counseling or counselling or counselor* or counsellor* or motivational interview* or directive therapy or ((behavior* or behaviour*) N2 (intervention or interventions OR therapy or therapies)))) OR (AB (counseling or counselling OR motivational interview* or directive therapy or ((behavior* or behaviour*) N2 (therapy or therapies))))	493
19	S17 OR S18	493

20	MM "Obesity+"	2
21	TI (overweight or obese or obesity) OR AB (overweight or obese or obesity)	197
22	S20 OR S21	197
23	S7 AND (S13 OR S16)	61
24	(S13 OR S16) AND S19	32
25	S22 AND S19	22
26	S23 OR S24 OR S25	98
27	((MM "Child+") OR (MM "Infant+") OR (MM "Adolescence+")) NOT ((MM "Adult+") OR (MM "Middle Age") OR (MM "Aged+") OR (MM "Young Adult"))	42
28	TI (adolescent* or teen or teens or teenager* OR child or childs OR children* or pediatric* or paediatric* OR nursery or newborn* or new born* or infant or infants)	1,330
29	AB (adolescent* or teen or teens or teenager* OR child or childs OR children* or pediatric* or paediatric* OR nursery or newborn* or new born* or infant or infants)	2,908
30	TI (schoolage* or school age* OR school based or school based or kindergar* or preschool* or pre-school* or elementary school* or junior high* or highschool*) or TI (K-12 N2 (student* or school* or education*))	32
31	AB (schoolage* or school age* OR school based or school based or kindergar* or preschool* or pre-school* or elementary school* or junior high* or highschool*) OR AB (K-12 N2 (student* or school* or education*))	114
32	MM ("Schools, Nursery")	1
33	S27 OR S28 OR S30 OR S31 OR S32	1,379
34	S26 NOT S33	81
35	S26 NOT S33	9

Cochrane Central

#	**QUERY {Cochrane Central Register of Controlled Trials, Ebsco; search date April 2, 2021]**	**Results**
1	(MM "Diet")	2,342
2	TI (diet or diets or dietary or nutrition*) OR AB (diet or diets)	62,655
3	MM "Eating"	413

4	TI eating	3,651
5	AB (eating habit or eating habits) OR AB (dietary intake OR dietary habit*)	10,588
6	AB ((healthy OR unhealthy or nutritional) N2 (eating or meal or meals))	2,573
7	S1 OR S2 OR S3 OR S4 OR S5 OR S6	70,872
8	(MM "Exercise+") OR (MM "Physical Fitness+")	11,515
9	TI (exercise or exercising or exercised or physical activity or physical activities OR aerobic activity or aerobic activities) OR TI ((aerobic* or fitness or pilates or yoga) N2 (class or classes))	55,323
10	AB (physical activity or physical activities or physical fitness or fitness routine or fitness class or fitness classes or aerobic exercis*) OR AB ((regular or daily or routine or weekly or week or weeks) N2 (exercis* or gym))	42,911
11	TI ((regular or daily or routine or weekly or week or weeks) N2 gym)	2
12	AB (aerobic activity or aerobic activities OR ((aerobic* or fitness or pilates or yoga) N2 (class or classes))	1,536
13	S8 OR S9 OR S10 OR S11 OR S12	78,196
14	MM "Muscle Strength+"	22,491
15	TI anthropomorphic* measur* OR AB anthropomorphic* measur*	60
16	S14 OR S15	60
17	((MM "Cognitive Therapy+") OR (MM "Behavior Therapy+")) OR ((MM "Motivational Interviewing") OR (MM "Peer Counseling") OR (MM "Anticipatory Guidance")) OR (MM "Nutritional Counseling")	706
18	(TI (counseling or counselling or counselor* or counsellor* or motivational interview* or directive therapy or ((behavior* or behaviour*) N2 (intervention or interventions OR therapy or therapies)))) OR (AB (counseling or counselling OR motivational interview* or directive therapy or ((behavior* or behaviour*) N2 (therapy or therapies))))	39,707
19	S17 OR S18	39,707
20	MM "Obesity+"	1,445
21	TI (overweight or obese or obesity) OR AB (overweight or obese or obesity)	44,932
22	S20 OR S21	44,932
23	S7 AND (S13 OR S16)	11,072
24	(S13 OR S16) AND S19	4,157
25	S22 AND S19	2,687
26	S23 OR S24 OR S25	15,383

27	((MM "Child+") OR (MM "Infant+") OR (MM "Adolescence+")) NOT ((MM "Adult+") OR (MM "Middle Age") OR (MM "Aged+") OR (MM "Young Adult"))	52,587
28	TI (adolescent* or teen or teens or teenager* OR child or childs OR children* or pediatric* or paediatric* OR nursery or newborn* or new born* or infant or infants)	129,966
29	AB (adolescent* or teen or teens or teenager* OR child or childs OR children* or pediatric* or paediatric* OR nursery or newborn* or new born* or infant or infants)	159,368
30	TI (schoolage* or school age* OR school based or school based or kindergar* or preschool* or pre-school* or elementary school* or junior high* or highschool*) or TI (K-12 N2 (student* or school* or education*))	6,809
31	AB (schoolage* or school age* OR school based or school based or kindergar* or preschool* or pre-school* or elementary school* or junior high* or highschool*) OR AB (K-12 N2 (student* or school* or education*))	12,197
32	MM ("Schools, Nursery")	146
33	S27 OR S28 OR S30 OR S31 OR S32	136,692
34	S26 NOT S33	13,483
35	S26 NOT S33	10,769
36	conference abstract	188,510
37	S35 NOT S36	9,120
38	S35 NOT S36	9,120
39	S35 NOT S36	9,120
40	S35 NOT S36	9,120
41	S35 NOT S36	9,120
